# “*It Is a Vicious Circle*”: Experiences of People Living With Obesity and Chronic Pain: A Qualitative Evidence Synthesis (QES)

**DOI:** 10.1111/obr.70004

**Published:** 2025-07-28

**Authors:** Natasha S. Hinwood, Maire‐Brid Casey, Colin G. Dunlevy, Catherine Doody, Catherine Blake, Bróna M. Fullen, Gráinne O'Donoghue, Susie Birney, Fionnuala Fildes, Keith M. Smart

**Affiliations:** ^1^ UCD School of Public Health, Physiotherapy and Sport Science University College Dublin Dublin Ireland; ^2^ UCD Center for Translational Pain Research University College Dublin Dublin Ireland; ^3^ Physiotherapy Department The Beacon Hospital Dublin Ireland; ^4^ TCD Discipline of Physiotherapy, School of Medicine Trinity College Dublin Dublin Ireland; ^5^ Center for Obesity Management St. Columcille's Hospital Dublin Ireland; ^6^ Irish Coalition for People Living With Obesity (ICPO) Dublin Ireland; ^7^ Independent Patient Insight Partner St. Vincent's Private Hospital Dublin Ireland

**Keywords:** chronic pain, CP, obesity, pain, protocol, qualitative evidence synthesis

## Abstract

**Introduction:**

The relationship between obesity and chronic pain (CP) is complex. Obesity is associated with increased pain‐related disability, pain intensity, worse physical functioning, and poorer psychological well‐being.

**Aims:**

The aim of this qualitative evidence synthesis (QES) was to systematically review and synthesize the qualitative literature reporting experiences of people living with obesity (PwO) and CP.

**Methods:**

We registered (PROSPERO: CRD42023361391) and undertook a QES to answer the following question: “What are the first‐person experiences of people living with obesity and chronic pain?”

We searched five databases on February 9, 2023 and February 8, 2024 to identify primary qualitative studies investigating the experience of PwO and CP. Two authors independently screened search results for eligibility, extracted data, assessed methodological quality using the Critical Appraisal Skills Program (CASP), and undertook a thematic synthesis of the included studies.

**Results:**

We included 10 studies (*n* = 153 participants) and identified eight findings under four main themes: (1) A Predominant Bio‐Mechanical Understanding of Pain; (2) Catch 22: Vicious Cycle of Pain and Obesity; (3) The Stigmas Associated With Pain and Obesity; and (4) Food as a Complex and Frustrating Pathway to Health.

**Conclusions:**

The lived experiences of CP and obesity include complex interactions of pain beliefs, challenges relating to healthcare provision, pain‐associated and weight‐related stigmas, altered self‐image, self‐blame, and altered food habits in response to pain. Our QES enhances understanding of experiences of PwO and CP and highlights the need for improved strategies for healthcare professionals to address weight‐based stigma and provide holistic care for PwO and CP.

**Abbreviations**
BMI: body mass index
CASP: critical appraisal skills program
CP: chronic pain
GRADE CERQual: Grading of Recommendations Assessment, Development, and Evaluation Confidence in Evidence from Reviews of Qualitative Research
MeSH: Medical Subject Headings
PICOS: Population, Intervention, Comparison, Outcome, and Study type
PPI: public patient involvement
PwO: people/person living with obesity

## 1 Introduction

Obesity is defined as a “*chronic, systemic illness characterised by alterations in the function of tissues, organs, the entire individual, or a combination thereof, due to excess adiposity*” [1]. It is a complex chronic disease in which excess or dysfunctional adiposity impairs health [1, 2]. Drivers and mechanisms for obesity are multifactorial and complex, involving genetic, epigenetic, environmental, behavioral, and socioeconomic factors [3, 4, 5, 6]. Prevalence of higher body weight is increasing worldwide [2, 7, 8]. Obesity also imposes large economic burdens nationally and individually [9, 10] and negatively impacts upon quality of life (including healthy life years) and mortality [11, 12]. People who live with obesity (PwO) experience increased pain incidence and severity [13]. Obesity is consistently and significantly associated with persistent pain and chronic pain (CP)—pain lasting ≥ 3 months [14]. Previous estimates indicated that 36.4% of PwO experience pain at least monthly, increasing up to 40% of people with body mass index (BMI) 35–39.9 kg/m^2^, when compared to 20% of people with BMI < 25 kg/m^2^ [15, 16]. An obesity registry study from Sweden previously reported up to 57.9% of men and 68% of women with obesity reporting pain in at least one of five locations, compared to 32.2% and 37% of men and women without obesity [17]. A retrospective analysis of data from an Irish Center for Obesity Management found that musculoskeletal (MSK) pain was reported in 91% of attendees (*N* = 915), including 69% reporting low back pain (LBP), of which the majority was classified as chronic LBP [18].

Mechanisms linking pain and obesity are complex and multifactorial and include genetic, behavioral (e.g., kinesiophobia), socioeconomic, cultural, biomechanical (e.g., decreased muscle mass in sarcopenic obesity or increased joint load), and physiological (e.g., inflammatory) components [19, 20, 21, 22, 23, 24, 25, 26, 27, 28, 29]. Pain is associated with poorer outcomes in physical functioning, including volume and complexity of physical movement, such as gait patterns (walking speed and motility) [16, 30, 31]. Co‐occurring pain and obesity have a worse impact on function and quality of life than either condition alone [32, 33], while integrating both obesity care and CP management leads to improved pain and disability compared with either intervention separately [18, 28, 34]. There is some low‐quality evidence suggesting that weight‐loss interventions can provide small‐to‐moderate improvements in pain and disability [33], including for people living with knee or hip osteoarthritis (OA) (when compared to minimal care) and low back pain [35, 36]. Some evidence suggests that the presence of obesity bias has negative effects on medical decision‐making and on the quality of care for PwO [37, 38, 39], while there is some low‐quality evidence to suggest that perceived weight‐related stigma also increases pain intensity for PwO [40, 41].

There is limited understanding of first‐person experiences of PwO living with CP. A mixed‐methods review, published in 2018, exploring the effectiveness and appropriateness of weight‐loss interventions for PwO and chronic musculoskeletal pain meta‐synthesized the findings from four qualitative studies (*n* = 88 participants) [42]. It identified a need (i) for healthcare professionals (HCPs) to understand the effects of pain on people's ability to control weight and (ii) to develop obesity management programs that address co‐occurring chronic musculoskeletal pain. This review included studies published prior to July 2016 and included people with chronic musculoskeletal pain only. An updated review that includes additional contemporary studies and participants with any type of chronic pain is necessary to enhance our understanding of the interaction between obesity and CP as well as identify potential knowledge gaps and shortfalls in existing care models [35, 43, 44, 45]. The aim of this qualitative evidence synthesis (QES) was to synthesize the available evidence exploring the first‐person experiences of people living with obesity and chronic pain.

## 2 Methods

This study was registered (PROSPERO registration ID: CRD42023361391), and methods reported in a published study protocol [46]. Five databases (MEDLINE, EMBASE, CINAHL, Web of Science, and PsycINFO) were searched to identify all first‐person perspective qualitative studies investigating the lived experience of PwO and CP. Two authors independently screened titles and abstracts and full texts for eligibility, extracted data, and undertook an iterative thematic synthesis of the results and discussions of the included studies [47]. Eligibility criteria were determined using a Modified PICOS structure (Table 1) [48]. Thematic synthesis was undertaken in three stages: (i) line‐by‐line coding; (ii) development of descriptive themes; and (iii) synthesis of analytical themes [46]. Two patient insight partners were involved in the synthesis of final themes, one with many years of lived experience of obesity and one with many years of lived experience with both obesity and chronic pain.

**TABLE 1:** Modified PICOS of inclusion and exclusion criteria.

	Inclusion	Exclusion
Population	People living with obesity (mean BMI ≥ 30 kg/m) People living with chronic pain (> 3 months) or any chronic musculoskeletal pain condition Adults (18 years and over)	People without experience of obesity (mean BMI < 30 kg/m) Studies involving children (17 years of age or younger) People without chronic pain People with cancer pain or pregnancy‐related pain
Intervention	Not applicable	Not applicable
Context	Any country or setting (e.g., primary, secondary, or tertiary care, and general population)	Perspective of experts working in research area
Outcome	Peoples' lived experience of pain	Experiences and opinions of professionals working with people with obesity
Study type	Qualitative, including interviews, focus groups, life histories, ethnographic studies, phenomenological studies, grounded theory studies, historical studies, case studies, and thematic analysis Focused on personal experience Original research	Studies that employ quantitative methods only

The methodological quality of the included studies was assessed using the Critical Appraisal Skills Program (CASP), and our confidence in the findings was assessed using the Grading of Recommendations Assessment, Development, and Evaluation Confidence in Evidence from Reviews of Qualitative Research (GRADE‐CERQual) approach [49, 50]. Findings were reported in accordance with the Enhancing Transparency in Reporting the Synthesis of Qualitative Research (ENTREQ) statement (Data S1) [51].

## 3 Deviations From Protocol

While inclusion only of participants with a BMI ≥ 30 kg/m^2^ was initially planned and outlined in the study protocol, a post hoc decision was made to include studies where the mean BMI of participants was ≥ 30 kg/m^2^. Papers that included people with BMI < 30 kg/m^2^ but failed to report mean BMI were excluded. This resulted in the inclusion of five papers from four studies that included some participants with BMI < 30 kg/m^2^ but with a mean BMI of included participants ≥ 30 kg/m^2^. Also, we excluded studies including people with cancer and pregnancy‐related pain because of the complexities and specificities of both conditions. The decision to amend our inclusion and exclusion criteria as described previously was to improve the quality of the data in this review by including a greater spectrum of data and quantity of studies with direct relevance to answering the research question, while respecting the necessary methodological transparency and repeatability [52]. Additionally, we made the decision to use the software package, NVivo 20 (Lumivero, Denver, CO, USA), for reference and data management. Finally, data richness was assessed using an evidence‐based data richness and thickness assessment tool developed for use with QESs [53]. These decisions were made to facilitate data management and were in‐keeping with similar QES in our field of research.

## 4 Results

**FIGURE 1:**
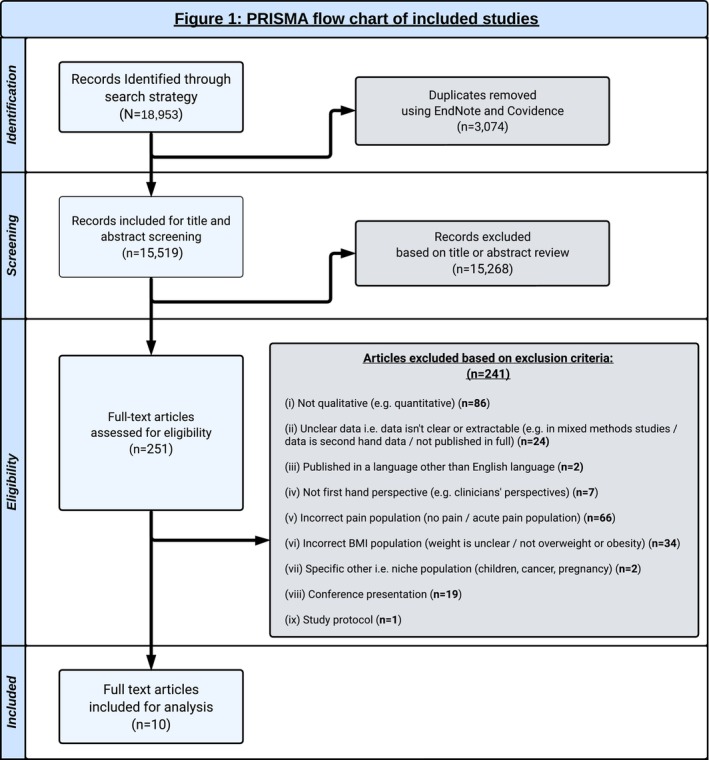
PRISMA flowchart of the included studies.

Databases were searched from inception to February 9, 2023. An updated search was conducted on February 8, 2024.The combined searches yielded 18,953 results, and after de‐duplication, 15,519 were screened. Notably, 251 full‐text studies were screened, of which 10 met our inclusion criteria and were included in the QES. Our study selection process, including reasons for exclusion of full texts, is detailed in Figure 1. Selected characteristics of the included studies are detailed in Tables 2 and 3. Full characteristics of the included studies can be found in Data S2.

**TABLE 2:** Participant sociodemographic, BMI, and pain characteristics of the included studies.

Study	Sociodemographic				BMI (kg/m^2^)		Pain description
	Age	Gender	Ethnicity	Employment status *n* (%)	Mean (SD)	Range	
Janke and Kozak 2012 (United States)	86.6% (26 of 30) age ≥ 50	*n* = 24 (of 30) (80%) male	73.3% (22 of 30) White	Unemployed or disabled, not retired 46.6% (14 of 30); retired 43.3% (13 of 30)	36.8 (8.9)	≥ 25	Average pain intensity 5.6 (SD 1.9); average pain interference 3.6 (SD 2.1); weekly pain at an intensity ≥ 4 (0 = none, 10 = worst imaginable) during the previous 3 months; current diagnosis of a medical complaint associated with persistent pain (e.g., osteoarthritis)
Bunzil et al. 2019 (Australia)	Mean 68 years; range 50–80+	*n* = 13 (of 27) (48%) female	Not reported	Not reported	33 (not reported)	19–30+; Overweight (11), PwO (16)	End‐stage knee OA, awaiting TKR
Cooper et al. 2018 (United Kingdom)	29–71; mean 53.5 (SD 11)	*n* = 16 (of 18) (89%) female	Not reported	Not reported	33.27 (5.94)	25–45.9; Overweight (6), PwO (12)	Mean pain score was 4.6 (out of 10) for intensity. Inclusion criterion was self‐reported persistent musculoskeletal pain ≥ 4/10 (0 = no pain, 10 = worst pain) any site for > 3 months. Participants reported pain in various sites, but particularly in their knees and lower back, with several reporting pain in multiple sites. Most of the participants reported that pain affected their enjoyment of life and ability to sleep. Several felt that it affected their relationships with others.
Craft et al. 2015 (United States)	Mean 50.8 (SD 6.3)	*N* = 15 (100%) female	Race, White, *N* (%) 14 (93.3)	Employed full time 8(53.3); part‐time 3(20.0); unemployed 3(20.0); homemaker 1(0.1)	37.88 (4.87)	≥ 30	Diagnosis of fibromyalgia (FM). The mean total score on the Fibromyalgia Impact Questionnaire (FIQR) was 56.5 (SD 12.5), indicating a high‐moderate effect of symptoms overall. Individual mean scores on the Patient Health Questionnaire (PHQ‐9) ranged from 2 to 19 (12.9–4.9), indicating moderate depressive symptomology.
Godziuk et al. 2022 (Canada)	Mean age 54.7 years (SD 9.7); age range 41–68	*N* = 20 (100%) female	Not reported	Work full time or part time 15 (75); on disability leave 2 (10); retired, laid‐off, unreported 3 (15)	Not reported	≥ 35	Self‐reported knee OA, predominantly reported bilateral knee OA (75%), symptom onset ≥ 5 years prior (85%); self‐reported severity: mild 4 (20), moderate 9 (45), severe 7 (35)
Janke et al. 2015 (United States)	86.6% (26 of 30) age ≥ 50	*n* = 24 (of 30) (80%) male	73.3% (22 of 30) White	Unemployed or disabled, not retired 46.6% (14 of 30); retired 43.3% (13 of 30)	36.8 (8.9)	≥25	Average pain intensity 5.6 (SD 1.9); average pain interference 3.6 (SD 2.1); weekly pain at an intensity ≥4 (0 = none, 10 = worst imaginable) during the previous 3 months; current diagnosis of a medical complaint associated with persistent pain (e.g., osteoarthritis)
O'Neill and Worboys 2011, (United Kingdom)	66 years old	*N* = 1 male	Not reported	Not reported	Not reported	BMI > 30	Pain associated with 10‐year history of lymphoedema
Toye et al. 2018 (United Kingdom)	Aged 59–76 years	*N* = 6 (100%) male	All White (British)	Employed = 4; retired = 2	33 (not reported)	31–38	Knee OA, awaiting knee joint replacement
Storm et al. 2023 (Sweden)	Mean 43.8 (SD 10.2); range 28–63	*n* = 11 (69%) female, *n* = 5 (31%) male	All Swedish	Employed = 11	35.7 (4.4)	30–43	Pain intensity (scale 0–10), Mean ± SD 5.5 ± 1.7 2.5–8; Pain duration, years, median (Q1–Q3) 8 (4.4–20.8); pain diagnosis: Fibromyalgia/widespread pain (5), lower back pain (4), hypermobility syndromes (4), joint pain (2), Myalgia (not specified) (1)
Lawford et al. 2023 (Australia)	Mean 65 years (SD 9.0)	*n* = 10 (50%) female, *n* = 10 (50%) male	Not reported	Not reported	32.7 (2.9)	28.1–37.9	Knee pain at baseline mean 5.6 (SD 1.4); knee pain at 6 months post‐intervention 1.6 (SD 1.3); history of knee pain on most days for at least 3 months and met OA criteria

**TABLE 3:** Characteristics of the included studies.

Study (country)	Study aim	Sample size	Methods							Findings
			Design	Setting	Sampling	Recruitment	Inclusion	Exclusion	Analysis	
Janke and Kozak 2012 (United States)	To identify perceptions of people with overweight/PwO and CMP regarding experiences with (i) barriers and facilitators for treatment; (ii) engagement with health‐promoting behaviors; and (iii) treatment for weight and/or pain control.	30	Either individual interviews or focus groups (< 4) with semi‐structured discussion.	Primary care clinics large Midwestern Veteran's Affairs (VA) hospital.	Purposeful.	Flyers posted in hospital common areas and waiting rooms and direct referral from providers.	(i) BMI ≥ 25 kg/m^2^; (ii) weekly pain at an intensity > 4 (0 = none, 10 = worst imaginable) during the previous 3 months; and (iii) current diagnosis of a medical complaint associated with persistent pain (e.g., osteoarthritis).	(i) Individuals < 18 years of age, (ii) inpatients, and those with (iv) difficulty communicating in English, (v) active substance abuse or (vi) pain exclusively cancer related.	Constant comparative method.	(i) Role of depression; (ii) pain, shame, and hedonic hunger; (iii) emotional eating and pain; (iv) altered dietary choices; and (v) reduced engagement in and low self‐efficacy for physical activity
Bunzil et al. 2019 (Australia)	To investigate the patient‐related factors that affect treatment decision, such as (i) beliefs/attitudes toward knee OA; (ii) beliefs/attitudes toward treatment; (iii) health system‐related factors (access and referral pathways).	27	Individual interviews (in‐person and phone) using an interview guide.	Orthopedic clinic of a large tertiary hospital in a metropolitan region of Australia.	Not reported.	Patients on the waiting list for TKA attending the orthopedic preadmission clinic were approached by a research assistant and invited to participate.	People older than 18 years, spoke English, had a diagnosis of knee OA, and had consented to undergo primary TKA. Nil BMI restrictions.	Patients were ineligible if they needed an interpreter or were unable to provide independent informed consent for TKA because of cognitive impairment.	Analysis using the Five‐Step Framework approach, informed by health belief model (common sense model).	(i) Identity belief: “*Knee OA is bone on bone”*; (ii) causal belief: “OA is due to excessive loading through the knee”; (iii) consequence beliefs: “Fear of falling and damaging the joint”; (iv) timeline beliefs: “OA as a downward trajectory”; “The urgency to do something”; “Arriving at the end of the road”; and (v) treatment beliefs: “The weight loss dilemma”; “Physiotherapy cannot help bone on bone”; “Replacing the cartilage”; “A mechanical problem requires a mechanical fix”
Cooper et al. 2018 (United Kingdom)	To gain insight into the lived experience of adults with overweight/obesity and CMP.	18	Face‐to‐face in‐depth, semi‐structured interviews.	Participants recruited from regional branches of a commercial weight loss service.	Purposeful.	Direct recruiting by research team, following a presentation, with the opportunity to speak to the researcher privately.	People (i) currently attending the commercial weight loss service; (ii) aged ≥ 18 years; (iii) BMI ≥ 25 kg/m^2^; (iv) self‐reported persistent musculoskeletal pain ≥ 4/10 any site for > 3 months and occurring most days.	Pregnant or breastfeeding women and those who did not speak English were excluded.	Interpretive phenomenological analysis.	(i) Pain as a motivator and barrier to weight loss; (ii) fear of weight causing more damage; and (iii) activity, at least certain types of activity, is positive.
Craft et al. 2015 (United States)	To find out from women with fibromyalgia (FM), (i) their needs and preferences for, and (ii) barriers to participation in weight management programs.	15	Focus group discussion	Community dwelling, patients who previously attended a FM treatment program (FTP) within the prior year.	Not reported.	Invitation letter to participate, sent by post.	(i) Attendance in the fibromyalgia treatment program (FTP) within the prior year, (ii) BMI of ≥ 30 kg/m^2^, (iii) 30–60 years of age, (iv) diagnosis of FM based upon either or both of the American College of Rheumatology 19,901 and 201,014 criteria, (v) residing in Minnesota, (vi) agreed to the Minnesota Research Authorization, allowing contact for participation in studies, and (vii) female gender.	Not discussed.	Thematic analysis.	(i) There are complex connections between fibromyalgia, weight, and exercise; (ii) healthy eating can be burdensome and time consuming for women with fibromyalgia; (iii) emotions play a key role in these connections, and women often feel misunderstood; (iv) there is a need for specialized weight management programs specifically for women with fibromyalgia.
Godziuk et al. 2022 (Canada)	To incorporate perspectives of people with OA and BMI > 35 kg/m^2^ in the design and delivery of a multimodal intervention intended to prevent muscle loss.	20	After reading about a 12‐week intervention, participants answered anonymous open‐ended questions, with the option of a one‐on‐one semi‐structured interview afterwards.	Anonymous online members of the public.	Purposeful and voluntary sampling.	Anonymous electronic survey, distributed on the Obesity Canada (OC) website and social media.	(i) Participants ≥ 40 years old; (ii) had a BMI ≥ 35 kg/m^2^; (iii) and had been told by a doctor that they have arthritis or OA in one or both knees. All self‐reported.	Not discussed.	Framework analysis, with thematic analysis.	(i) Emphasize “health gains” over “weight loss”; (ii) support and language matter; (iii) include options for customization.
Janke et al. 2015 (United States)	To understand patients' (i) preferences and (ii) dissatisfaction with pain and obesity care in a primary care setting.	30	Either individual interviews or focus groups (< 4) with semi‐structured discussion.	Primary care clinics large Midwestern Veteran's Affairs (VA) hospital.	Purposeful.	Flyers posted in hospital common areas and waiting rooms and direct referral from providers.	(i) BMI ≥ 25 kg/m^2^; (ii) weekly pain at an intensity > 4 (0 = none, 10 = worst imaginable) during the previous 3 months; and (iii) current diagnosis of a medical complaint associated with persistent pain (e.g., osteoarthritis).	(i) Individuals < 18 years of age, (ii) inpatients, and those with (iv) difficulty communicating in English, (v) active substance abuse or (vi) pain exclusively cancer related.	Analysis used the constant comparative method.	(i) Need for information tailored to comorbidity; (ii) frustration with available treatment approaches and desire for motivation enhancement; (iii) provider initiated concern and communication
O'Neill and Worboys 2011 (United Kingdom)	To look at the patient journey and the challenges of a patient attending the service. The patient was a person living with obesity and lymphoedema.	1	A short interview with the patient on his experience as a patient living with lymphoedema.	The patient presented at an outpatient wound healing service.	Not reported.	Not discussed.	Not discussed.	Not discussed.	Nil structured analysis described.	That the patient's management was largely autonomously driven, which underscored the need for investment in related services.
Toye et al. 2018 (United Kingdom)	To explore barriers to weight loss in a group of older men with osteoarthritis.	6	Two sets of semi‐structured interviews with men who had undergone total knee replacement (TKR). One prior to joint replacement surgery and one 12‐month post‐op.	Participants interviewed at home (*n* = 5), or at the hospital (*n* = 1). Two patients were joined by their wives during the interviews.	Not reported.	Not specifically described. Men who underwent surgery (TKR).	Men who underwent surgery (TKR). Not discussed explicitly or in further detail.	Not discussed.	Analysis using a constructivist grounded theory approach.	There were tensions in men's body talk that might influence their healthcare decisions: “*I am big and healthy and do not need to lose weight,”* and yet “*being this size cannot be that good for me*.” Men discussed reasons that they might put on weight, which might be potential barriers to weight loss.
Storm et al. 2023 (Sweden)	To explore patient perspectives on how chronic pain and obesity influenced (i) each other; (ii) ability to make lifestyle changes.	16	Semi‐structured interviews, either by phone or in‐person at the pain clinics, using an interview guide.	Previous patients of an 8‐week Interdisciplinary Pain Rehabilitation Program in two specialist pain rehabilitation clinics in Sweden.	Purposeful.	Patients contacted consecutively from the Swedish Quality Registry for Pain Rehabilitation (SQRP) and invited to participate.	Clinic patients with (i) chronic pain and (ii) obesity (body mass index (BMI > 30 kg/m^2^), (iii) who had completed an IPRP between 2019 and 2021.	Not discussed.	Latent content analysis.	(i) Lifestyle changes are burdensome with a body broken by both pain and obesity; (ii) pain disturbing days and nights worsens weight control; (iii) pain‐related stress makes lifestyle changes harder; (iv) a body affected by pain and obesity, intertwined with negative emotions; and (v) the overlooked impact of obesity on chronic pain
Lawford et al. 2023 (Australia)	To explore experiences of people with knee OA who are aiming to maintain weight loss following a multicomponent remotely delivered, clinician‐supported weight loss program.	20	Nested qualitative study. Semi‐structured interviews conducted over the telephone with participants who had been randomized to the weight‐loss arm of an RCT and had successfully lost weight.	Community‐dwelling adults with private health insurance from across Australia.	Not reported.	Participants were invited 1–3 weeks of having completed their final 12‐month questionnaire for the RCT. Recruitment for RCT was done through the private health insurance company by targeted emails.	Previously participated in the RCT. Eligibility for the RCT: (i) held private health insurance with a specific insurer that included cover for arthroplasty surgery; (ii) met the National Institute for Health and Care Excellence OA clinical criteria (aged 45 years, activity‐related joint pain, morning stiffness 30 min); (ii) 10 had average knee pain 4 on 11‐point numeric rating scale (0 = no pain, 10 = worst pain possible) in the past week; (iv) had a history of knee pain on most days for at least 3 months; (v) were aged < 81 years; and (vi) had a BMI 28 kg/m^2^ and < 41 kg/m^2^.	Same as the exclusion criteria for the RCT, including recent knee surgery (6 months) or due surgery, unable to participate in VLCD. Full eligibility criteria reported in the trial protocol [54].	Inductive thematic analysis.	(i) Successfully maintained weight loss; (ii) empowering self‐management of weight (understanding importance of exercise and physical activity, increased knowledge about food and nutrition, resources from program still useful, knee pain as a motivator, have confidence in ability to self‐regulate weight; (iii) challenges keeping on track.

**FIGURE 2:**
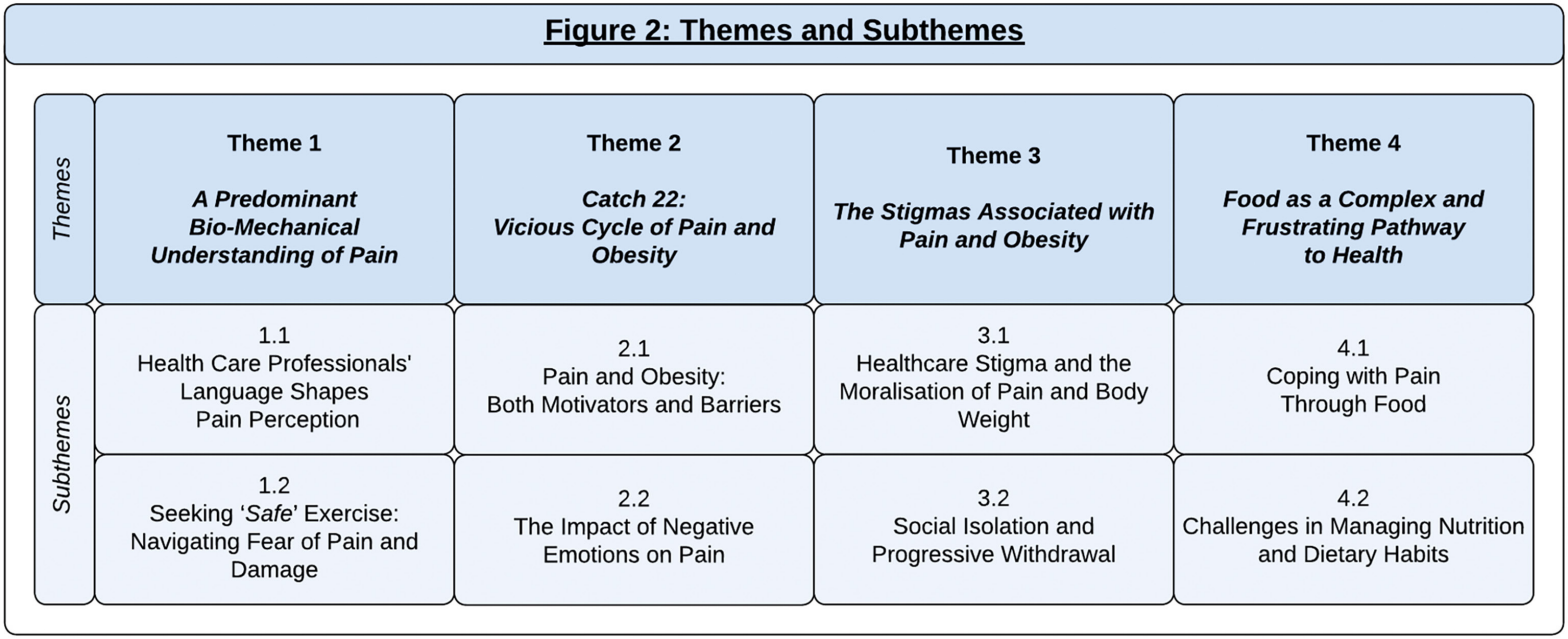
Themes and subthemes.

Included studies were uploaded to qualitative data management software NVivo 20. A thematic synthesis was conducted in three stages [47]. Stage 1 identified a total of 250 unique codes. In Stage 2, these codes were amalgamated thematically into an initial 36 themes. These themes were subsequently condensed again, through ongoing repeated dialogue and consensus, into 18 descriptive themes. In Stage 3, through additional iterative discussion and in consultation and discussion with two patient insight partners, the descriptive themes were further synthesized into four themes and eight subthemes (Figure 2; Data S3).

### 4.1 Summary of the Included Studies

Ten papers from nine individual studies were included (*n* = 153 participants; 65% female) (Table 2) [55, 56, 57, 58, 59, 60, 61, 62, 63, 64]. The findings from one study were published in two separate papers [55, 56]. Characteristics of researchers were not commonly reported but included physiotherapists, lymphoedema specialists, nurses, medical students, physicians, occupational therapists, and a psychologist, with varied previous expertise in qualitative research reported. Data collection was predominantly through individual semi‐structured interviews (*n* = 7), focus groups (*n* = 1), or a mixture of both (*n* = 1). Analysis methodologies included thematic analysis (*n* = 3), framework analysis (*n* = 2), the constant comparative method (*n* = 1), interpretive phenomenological analysis (*n* = 1), constructivist grounded theory (*n* = 1), and latent content analysis (*n* = 1).

All nine studies were from high‐income countries, with eight from countries with English as a first language (the United States, Canada, the United Kingdom, and Australia) and included predominantly ethnically White participants [55, 56, 57, 58, 59, 60, 61, 62, 64]; one study was from Sweden [63]. Sample sizes ranged from 1 to 30. Eight studies reported a mean BMI, which was used to calculate an overall mean BMI of 30.29 kg/m^2^ (BMI range: > 19 kg/m^2^, with nil upper limit specified). Half of the studies included people with a history of OA (*n* = 5), typically knee OA. Other pain conditions represented included fibromyalgia (FM)/widespread pain (*n* = 2), lymphoedema (*n* = 1), lower back pain (*n* = 2), hypermobility syndromes (*n* = 1), and myalgia (unspecified) (*n* = 1) (Table 3).

### 4.2 Methodological Quality of the Included Studies

Eight of the nine studies (i.e., 10 included papers from nine studies) were judged as being of moderate‐to‐high methodological quality, and one study was of very poor quality [61]. Only one of the included studies posed no methodological concerns [58], eight posed minor concerns regarding transparency, reflexivity, and ethical considerations [55, 56, 57, 59, 60, 62, 63, 64], and one study posed major concerns regarding ethical considerations, methodological rigor, transparency, and suitability [61]. Quality assessments of the included studies are presented in Table 4.

**TABLE 4:** Quality assessment (CASP) of the included studies.

Study	*1. Was there a clear statement of the aims of the research?*	*2. Is a qualitative methodology appropriate?*	*3. Was the research design appropriate to address the aims of the research?*	*4. Was the recruitment strategy appropriate to the aims of the research?*	*5. Was the data collected in a way that addressed the research issue?*	*6. Has the relationship between researcher and participants been adequately considered?*	*7. Have ethical issues been taken into consideration?*	*8. Was the data analysis sufficiently rigorous?*	*9. Is there a clear statement of findings?*	*10. How valuable is the research?*
Janke and Kozak 2012	Yes	Yes	Yes	Yes	Yes	Cannot tell	Yes	Yes	Yes	Yes
Bunzil et al. 2019	Yes	Yes	Yes	Yes	Yes	No	Yes	Yes	Yes	Yes
Cooper et al. 2018	Yes	Yes	Yes	Yes	Yes	Yes	Yes	Yes	Yes	Yes
Craft et al. 2015	Yes	Yes	Cannot Tell	Yes	Yes	Yes	Yes	Yes	Yes	Yes
Godziuk et al. 2022	Yes	Yes	Yes	Yes	Yes	Cannot tell	Yes	Yes	Yes	Yes
Janke et al. 2015	Yes	Yes	Yes	Yes	Yes	Cannot tell	Yes	Yes	Yes	Yes
O'Neill and Worboys 2011	Yes	Yes	No	No	No	Cannot tell	No	No	No	No
Toye et al. 2018	Yes	Yes	Yes	Cannot Tell	Yes	Cannot tell	Yes	Yes	Yes	Yes
Storm et al. 2023	Yes	Yes	Yes	Yes	Yes	Cannot tell	Yes	Yes	Yes	Yes
Lawford et al. 2023	Yes	Yes	Yes	Yes	Yes	Cannot tell	Yes	Yes	Yes	Yes

### 4.3 Themes and Subthemes

We identified four main themes and eight subthemes (Figure 2), including the following: (1) *A Predominant Bio‐Mechanical Understanding of Pain*; Subtheme 1.1: *Healthcare Professionals' Language Shapes Understanding;* Subtheme 1.2: *Seeking “Safe” Exercise: Navigating Fear of Pain and Damage*; (2) *Catch 22: Vicious Cycle of Pain and Obesity;* Subtheme 2.1: *Pain and Obesity: Both Motivators and Barriers*; Subtheme 2.2: *The Impact of Negative Emotions on Pain*; (3) *The Stigmas Associated With Pain and Obesity*; Subtheme 3.1: *Healthcare Stigma and the Moralization of Pain and Body Weight*; Subtheme 3.2: *Social Isolation and Progressive Withdrawal*; and (4) *Food as a Complex and Frustrating Pathway to Health*; Subtheme 4.1: *Coping With Pain Through Food*; Subtheme 4.2: *Challenges Managing Nutrition and Dietary Habits*. These themes are discussed next, with supporting data available in Data S3–S5.

#### 4.3.1 A Predominant Bio‐Mechanical Understanding of Pain

One of the most striking themes present throughout the included texts were how participants often described their own pain in terms of a biomechanical understanding. These pain‐associated beliefs were illustrated typically using descriptors like “*wear and tear,”* “*bone on bone,” or “crumbling.”* This was contextualized in a common experience of directly associating pain with movement. These clustered beliefs related to how participants perceived pain in relation to movement and exercise and included the following: kinesiophobia (fear of movement) due to perceived potential damage; perceiving joint loading as damaging; lack of confidence in their own body to perform basic physical activities, such as walking, because of pain and pain‐associated fatigue; weight gain as the cause of pain; that weight needed to decrease to improve pain (but also conversely that pain needs to improve to lose weight); that tissue or bodily degradation was inevitable because of age; only mechanical solutions and external factors (i.e., surgery) would resolve the pain; and finally, a sense of futility regarding personal ability to influence or self‐manage pain and weight. Much of this understanding was represented by those with experience of OA.

*“Well what's the point in trying to do something when something's worn out? I believe in nuts and bolts; if something's worn out, you pull it out and put a new part in.”* (Bunzil 2019)

##### 4.3.1.1 Healthcare Professionals' Language Shapes Pain Perception

Central to patients understanding of their own pain through a biomechanical lens was the predominantly biomechanically oriented language used by HCPs. As proposed by researchers in one study [58] and supported by data from several others [55, 56, 60], potentially careless or inappropriate use of biomechanically focused language from HCP led to misinterpretation, inadvertently reinforcing negative or inaccurate beliefs in participants. This was particularly noticeable in the case of fear‐related pain beliefs such as kinesiophobia and low confidence about bodily integrity. Examples of this include concepts from HCPs that conveyed futility and language such as “*bone on bone*” or “*crumbling*” when describing anatomical changes.

*“I've got a collapsing spine,” While Emily was not surprised when she developed arthritis in her knees, as she had already been warned that this was likely to occur: “it must be about ten year ago, something like that. An’ I always remember the girls sayin’, the nurse sayin’‐ when I went for ma physio [for back pain] that the next thing to go would probably be ma knees”* (Cooper 2018)

Participants suggested how information from HCPs supported a foundational biomechanical understanding of their pain. Participants appeared to trust the information from the HCPs even in cases where they reported feeling emotionally unsupported or implied an overall negative or stigmatizing interaction with the HCP.

##### 4.3.1.2 Seeking “Safe” Exercise: Navigating Fear of Pain and Damage

Associated with this biomechanical understanding of pain were some kinesiophobic beliefs. These fear‐based beliefs were based on the biomechanical understanding of pain that perceived pain as an indicator of tissue damage resulting from overuse. Pain vigilance and perceived risk of causing further damage from movement were apparent barriers to exercise. However, many also expressed a desire to engage in exercise despite previous perceived failed attempts and ongoing fear associated with movement. Participants expressed an appreciation for the psychological and physical benefits of exercise, and how positive mood and small gains had an empowering effect more widely on self‐efficacy and motivation. Participants expressed a need for “*safety*” during exercise not only in terms of type and quantity but also in terms of psychological support. Exercise types that involved reduced biomechanical joint loading, such as swimming or cycling, were perceived as safer.

Participants expressed a need for support with managing the perceived threat of movement, regulating mood, and engaging with exercise, but also that this support needed to come from sources who understood their comorbidities and complexity (either peers or HCPs). It was clear that in discussing “*safety,*” participants were discussing holistic patient‐centered care and finding exercises and activities that were manageable with both conditions, that is, pain and obesity, while simultaneously addressing psychological care needs.

*“Ellie found some forms of exercise resulted in pain and swelling of her joints, and considered cycling as a safe form of exercise: ‘I was quite up f'for gettin’ a bike ‘cos I thought “well that'll ease the pressure on ma knees an’ ma ankles if I can cycle.” However, when she went to buy a bike, concern that she was too heavy for the tyres resulted in her leaving the shop without buying or even approaching a sales assistant, who may have been able to help. Her embarrassment about her size was evident in the way she paused and laughed when she said “there wasn't a tyre that could… take ma weight ((laughing)) on any of the bikes so*.*. ..”* (Cooper 2018)

In addition, participants expressed a need for this support for managing both their obesity and pain to be long term as they felt the current supports in place to be temporary and felt easily dismissed by HCPs. Others sought surgical solutions for their pain, based on a predominant biomechanical understanding of their pain (Theme 1).

#### 4.3.2 Catch 22: The Vicious Cycle of Pain and Obesity

This theme reflects the frustration stemming from the relentlessness and cyclical nature of trying to manage both chronic pain and obesity simultaneously. Many participants described their pain in terms of how it negatively impacted specifically their weight and vice versa.

*“It is a vicious circle of.. .. . . y'know tryin' to lose weight, tryin' to.. … y'know not be in pain, tryin' to exercise and then be in pain…. . ..y'know”* (Cooper 2018)

##### 4.3.2.1 Subtheme 2.1: Pain and Obesity: Both Motivators and Barriers

The relationship between pain and obesity was described heterogeneously by participants, with some believing that their pain was not caused by their body size (e.g., pain as due to previously active lifestyles). Most commonly, they saw obesity as an aggravating factor. Body weight was perceived as both a primary source of pain and as a barrier to improving pain. For these participants, weight loss was perceived as central to their pain management strategies. Conversely, participants largely expressed frustration that pain was a barrier to weight loss. Participants in nearly all studies discussed the frustration and sense of despair stemming from this paradox, with varying descriptors relating to this “*vicious cycle*.”

*“It [pain] took away the desire to lose weight … I was just like, ‘Oh, forget it. I do not want to worry about that right now.”* (Janke 2012)

*“I kayak every week, I play golf every week … And then I was doing my exercises that the physio gave me, and I was doing them probably, on average, once a week. But my knee's been so good the last couple of months I've really stopped doing that as well, so yeah.”* (Lawford et al. 2023)

Some participants also described pain as a motivating factor for physical activity, feeling improved pain and mobility with increased physical activity. However, many participants also expressed frustration and perceived poor success with trying to manage both pain and obesity through mechanisms focused mainly on weight loss, such as dieting or engaging with exercise. For some participants, their focus on weight loss was discussed as a central goal, while for others, their focus was more specifically on improving their chronic pain. Many participants also highlighted additional aggravating factors that further compounded the difficulties of managing their obesity and chronic pain. These included intrinsic factors, such as managing other health conditions, as well as extrinsic barriers like a lack of access to appropriate equipment designed for PwO and systemic factors associated with health care (limited treatment options, poor access to care, and financial barriers).

*“One of the worst parts about it is that if I was more active, I could lose a bit of weight and take weight off [the knee]. But you're buggered because you can't do something as simple as walk down the street.”* (Bunzil 2019)

##### 4.3.2.2 Subtheme 2.2: The Impact of Negative Emotions on Pain

In relation to managing both pain and obesity simultaneously, one of the intrinsic factors that was most apparent for PwO and CP was the central role of mental health and the impact of difficult negative emotions. Studies highlighted how negative emotions influenced participants' experiences of living with both pain and obesity and how there was a perceived lack of support for mental health. For example, many participants referred to feeling “*depressed*” and even more to low mood. Because of the amplifying effect of low mood on pain, this was commonly described as a significant barrier to improving well‐being. Many also described how low mood added to feelings of fatigue, which undermined motivation and self‐efficacy toward adopting and maintaining healthy behaviors and routines. This, in turn, sometimes led to further negative feelings such as shame and a personal sense of failure. Overall, the impact of negative emotions amplified the cyclical nature of trying to manage both chronic pain and obesity simultaneously.

“*The depression just makes it all that much worse. I think the treatments for pain will work better if we could get depression under control.”* (Janke 2012)

Some participants discussed challenges in coping with low mood, with some participants blaming antidepressant medication for further weight gain. Many highlighted a lack of support and the need for a whole‐person approach for pain and obesity treatments that take this into consideration, with specific provision for emotional support (either through peer support or from a HCP as needed).

#### 4.3.3 The Stigmas Associated With Pain and Obesity

Studies identified various types of stigmas encountered by people living with pain and obesity. Stigma was described in both healthcare and social settings (e.g., in clinical settings with HCPs, at home, and interactions in public places) and included weight bias, pain stigma, and social judgment. Elements of self‐stigma were also frequently described, with an altered sense of self‐image that resulted from living with pain and obesity.

*“I'm tired of ‘it's in your head, you're too lazy’ and then the doctors that don't want to take you on because you are the problem patient.”* (Craft 2015)

##### 4.3.3.1 Subtheme 3.1: Healthcare Stigma and the Moralization of Pain and Body Weight

Weight bias was frequently described and commonly within the context of health care and HCPs. Participants in seven studies highlighted what they felt to have a lack of understanding and holistic support from HCPs with respect to addressing their pain and weight needs. They described a sense of blame emanating from HCPs for both weight and pain. Some participants described other health conditions being attributed to weight. In one study, a participant recounted denial of treatment contingent on independently losing weight. At times, weight stigma and the moralization of weight loss were described in the context of what participants felt were unrealistic treatment plans prescribed by HCPs in a non‐collaborative manner, suggesting a lack of understanding and compassion more broadly. This intensified participants' own feelings of personal responsibility and subsequent sense of personal failure resulting from pain flare‐ups or weight gain.

*“‘You got to lose some weight’. [to have less pain] The first guy I told you about, he handed me a sheet that was 1200 calories a day. He said ‘Follow this; you'll lose weight. Don't come back here if you haven't lost 20 pounds’. The first month, I lost 20 pounds, the next month I only lost 18 pounds, and he just tore me apart …”* (Janke 2015)

Some participants described pain stigma in the form of pain minimization or dismissal, which was often coupled with weight bias and attributed to the participants' body size. Elements of moral judgments based on pain behaviors and experiences were also present in the included studies. Some participants associated pain with morally positive or negative behaviors, such as attributing pain to many years of “*hard work*.” Low pain tolerance and/or expressing pain were also variously framed in morally negative terms.

*“Look, it's wear and tear … I expected this, I'm a hard worker,” Participant 15, man, 79 years old; “I knew I was going to get it because every second person that works in the yard has got arthritis because of the way we work.”* (Janke 2012)

##### 4.3.3.2 Subtheme 3.2: Social Isolation and Progressive Withdrawal

Participants also described elements of social isolation and progressive social withdrawal. They perceived a lack of understanding from family at times and explained how living with co‐occurring CP and obesity negatively affected engagement with many aspects of daily life, which cumulatively had a progressively reductive impact psychologically and socially. This was described in several ways, such as not being able to engage with activities that had been previously manageable, both inside and outside the home; feeling discouraged from engaging with physical activity in a public setting; a lack of understanding from family members and HCPs; and a progressive sense of social isolation. This sense of isolation appears to inform internalized narratives relating to internalized shame, self‐blame, and personal failure. Participants described challenges engaging socially because of a combination of anticipated social judgment and self‐blame and how these factors worked in combination to exhaust self‐efficacy and debase positive self‐perception.

*“My husband is like, ‘Really? You don't want to get up off the couch?’ And I say, ‘Honey, I just can't function’.”* (Craft 2015)

Participants frequently reported a sense of personal failure and frustration linked to their perceived limited weight loss. Self‐blame and personal failure were common elements of self‐stigma described by participants of the included studies. PwO and CP personalized responsibility for a perceived loss of self‐control over internal impulses, such as eating in response to hunger or lack of impetus for physical activity in the context of fatigue. This perceived moral failing stemmed from a commonly established misperception of “*mind over matter*” in relation to controlling obesity primarily, with pain as a secondary implied consequence. This, in the context of beliefs around their own body limitations and the unpredictability of pain, led to a sense of lack of control, social isolation, and altered self‐image. These experiences could be a likely manifestation of external stigma and internalized weight bias, contributing to a sense of marginalization and isolation.

*“I love to pick up whatever I want to eat, fix it and eat it. But, I know now that I can't because if I do, I'm going to keep getting bigger and bigger and bigger. And, that right there, in itself, plays the emotional part. Because when I'm standing in the bathroom, getting ready to get in the shower, I look at myself and I cry. And I think, ‘How did I get here?’ So, it all plays a role but then I'm hurting, so I don't want to go downstairs and do exercises … But my emotional comes back on the pain and if I was smaller, maybe I wouldn't hurt as bad to where I could get in 30 minutes every other day of exercise.”* (Craft et al. 2015)

#### 4.3.4 Food as a Complex and Frustrating Pathway to Health

This theme represents how participants depicted their relationships with food. Participants commonly reported altered responses and habits in relation to food choices and consumption as a result of pain. Participants perceived food as key to improving health and managing both their pain and obesity. Participants expressed a typically intrinsic desire for changes to dietary habits but frustration with perceived failure at sustainable change despite multiple previous attempts. Finally, some participants expressed either positive or negative moral framing of certain food behaviors, such as eating large volumes of foods or certain food types.

##### 4.3.4.1 Subtheme 4.1: Coping With Pain Through Food

In some studies, strong emotional elements and some moral attributes were associated with food and eating. Altered food habits were described both as a direct comfort‐seeking response to pain and as an indirect consequence of pain, because of pain disrupting habits and daily routines. PwO and pain described an increased frequency and volume of food consumption as a coping mechanism and response to both the experience of pain and the emotional distress associated with pain, sometimes in the absence of hunger. While participants recognized the increased use of food for self‐soothing, they often also experienced increased frustration, distress, and a sense of personal failure as a result. This behavior was variously described as “*food addiction*” or “*binges*” by participants or study authors.

*“In no way can I be hungry … I use food to soothe myself, but it really doesn't because once I've overeaten I say ‘Well, there. You did it again.’ I sabotage myself.”* (Janke 2012)

Participants also reported a change in the quality of nutrition when experiencing pain, opting for foods with a higher calorie and sugar content to self‐soothe and manage fatigue. The relationship between living with both pain and obesity and living with fatigue was an important consideration for many participants in the included studies, as many associated their increased fatigue with an inverse ability to plan and prepare food, opting for foods that were faster and easier to prepare.

*“It [pain] took away the desire to lose weight … I was just like, ‘Oh, forget it. I don't want to worry about that right now.’ You're just so worn out, the only thing you can think to do is give yourself a sugar kick and try to get something done.”* (Storm 2023)

The concept of hedonistic eating was also present in these altered food responses to pain. This was primarily eating as a source of pleasure in the absence of other sources because of reported daily living restrictions and unpredictability of pain. These daily living restrictions have previously been discussed but included increased time spent at home and reduced time engaging in activities outside the home (such as work or socializing). This provided increased opportunity for eating in the absence of other activities.

##### 4.3.4.2 Subtheme 4.2: Challenges in Managing Nutrition and Dietary Habits

Participants described “healthy” dietary habits as a key to managing their pain and obesity. Some participants reported sometimes multiple attempts at altering dietary patterns. These were experienced with mixed success but typically perceived as failed attempts by participants. Attempts at altering dietary patterns were also described as unsustainable, and participants expressed skepticism regarding practicality of altering food habits in the medium to long term, such as in the case of low‐calorie diets (LCD). In the context of socializing and relationships, PwO reported difficulty maintaining habits and motivation for routines perceived as healthy. These difficulties were perceived with a sense of doubling the sacrifice and isolation further to those already experienced (i.e., not being able to “*indulge*” in the food and also not being able to have the same freedom of choice or experiences as others). Conversely, those who had participated in interventions involving LCDs reported success following the intervention in response to improved knowledge and mindfulness related to food consumption.

*“I've probably learnt one thing, is not to overindulge in your meal; like the size of a meal. And I'm probably a lot more cognizant of the types of food I've got on my plate. So I try and avoid any real fatty items and I really only just have vegetables or salad with my dinner, and just whatever bit of meat or fish or something. So I am aware of not pigging out, if you like, on quantities of food and trying to keep them reasonably well.”* (Lawford 2024)

### 4.4 Confidence in the Review Findings

Confidence in the review findings was assessed using the GRADE‐CER‐Qual approach (Data S3) [50, 65, 66]. Overall, there was high confidence in three main themes and five subthemes (*Theme 1: A Predominant Bio‐Mechanical Understanding of Pain; Subthemes 1.2: Seeking “Safe” Exercise: Navigating Fear of Pain and Damage; and 2.2: The Impact of Negative Emotions on Pain; Theme 3: The Stigmas of Pain and Obesity; Subthemes 3.1: Healthcare Stigma and the Moralization of Pain and Body Weight; and 3.2: Social Isolation and Progressive Withdrawal; Theme 4: Food as a Complex and Frustrating Pathway to Health; Subtheme 4.1: Coping With Pain Through Food*).

We had moderate confidence in one theme and three subthemes *(Subtheme 1.1: Healthcare Professionals' Language Shapes Understanding; Theme 2: Catch 22: Vicious Cycle of Pain and Obesity; Subthemes 2.1: Pain and Obesity as Both Motivators and Barriers; and 4.2: Challenges Managing Nutrition and Dietary Habits)*. Findings with only moderate confidence should be interpreted with caution because of limited data richness, absence of supportive data in other papers, and moderate concerns regarding the methodological rigor, adequacy, and relevance of some of the papers that contributed to these findings.

## 5 Discussion

We identified four main themes associated with people's experiences of living with obesity and pain, including the following: (1) *A Predominant Bio‐Mechanical Understanding of Pain*; (2) *Catch 22: Vicious Cycle of Pain and Obesity*; (3) *The Stigmas Associated With Pain and Obesity*; and (4) *Food as a Complex and Frustrating Pathway to Health*.

Each theme reflects a dynamic and complex relationship between the lived experiences of pain and obesity. These experiences are shaped by both internal (intrinsic coping mechanisms; the role of mental health as a barrier; threat vigilance; and self‐perception and internalized weight stigma) and external factors (weight stigma and healthcare bias; social judgment; and barriers to education or opportunities to contextualize physiological signs and symptoms).

The findings from our QES are similar to others that have separately investigated people's experiences of living with CP and obesity [47, 67, 68, 69]. For example, people living with CP have described the rise of negative emotions; pain‐associated social isolation, reduced quality of life and an altered sense of self; and associated shame [67]. PwO have similarly described the life‐altering effects of obesity and experiences of weight and healthcare stigma, social judgment, shame, and blame [39, 69]. Our QES provides additional insights into the progressive and persistent way that obesity and co‐occurring CP restrict and frustrate patients' self‐efficacy and debase a sense of positive identity. This review also enhances current understanding of how obesity and CP co‐occurrence undermines patients' efforts to improve their own quality of life and engage with healthful routines, particularly regarding dietary habits and engaging in physical activity. We identified three novel conceptual insights, including (i) a predominant biomechanical understanding of pain in PwO and CP; (ii) the importance of language used by HCPs in terms of framing PwO biomechanical pain beliefs; and (iii) threat perception and significance of “*safety*” for PwO and CP.

Improving HCPs awareness regarding the experiences of PwO and using a person‐centered approach to the treatment of obesity were recommendations of these previous reviews [39, 42, 67, 68, 69]. An additional previous mixed‐methods review that explored weight‐loss interventions only for PwO and CP also had recommendations for holistic management of both conditions concurrently [42].

Participants' predominantly biomechanical understanding of pain is at odds with contemporary pain science and practice that acknowledges the broader multidimensional biopsychosocial determinants of the pain experience and the need for multimodal pain treatment and care [70, 71, 72]. Furthermore, language employed by HCPs may be central to this predominant biomechanical understanding of pain and inadvertently support unhelpful pain beliefs. However, pain science education in healthcare programs is limited [73]. This suggests that improved pain science education for HCPs may better help them support patients to address unhelpful pain beliefs. Thus, addressing the predominant biomechanical‐based beliefs held by PwO and chronic pain through biopsychosocially oriented pain science education, for both HCPs and patients alike, may contribute to improved understanding and management of pain [70, 74].

The mechanisms of and interventions for comfort eating, also called “emotional eating” or “hedonic eating,” have been discussed [75, 76, 77, 78]. Comfort eating is commonly defined as responding to negative feelings for temporary comfort by overconsuming energy‐dense and palatable foods, with mechanisms involving confusing internal states of hunger and satiety with physiological changes related to emotions [71, 72, 75]. While to date no systematic review has examined comfort eating in PwO and CP, our findings highlighting coping with pain through food and the role of negative emotions for PwO and CP are consistent with those of a previous review examining emotional regulation in binge eating disorder and PwO [79], other evidence of emotional eating in people with CP [80], and eating‐associated analgesia [77, 81]. Our findings further highlight the central role of mood and the ongoing challenges of managing obesity and CP concurrently. The role of emotions in pain amplification or attenuation is widely known [82, 83]. Improving chronic pain using strategies that synergistically include emotional support and regulation could help improve food‐related routines. This could be both direct in terms of alleviating the driver for relief from pain and indirect in terms of alleviating a barrier to engaging with the external environment. However, strategies aimed at addressing movement habits need to be employed selectively and tactically.

The mantra of “*eat less, move more”* has, until recently, been the most dominant treatment narrative concerning PwO [2, 84]. This narrative has roots in framing obesity drivers as being predominantly under volitional control, or lack‐thereof, and attribution of obesity to personal responsibility ignores our understanding of obesity as a disease and has contributed to the stigmatization of PwO [85, 86]. The persistence of this mantra is reflected throughout the findings of this QES—in particular with respect to the moralization of pain and body weight. There have been consistent similar findings and discussions in relation to experiences of multiple types of stigmas in people with CP. Collectively, these include weight bias in health care, stigma in pain perception (pain dismissal or minimization), and societal judgment or discrimination. This QES adds further evidence to the dual burden of stigma experiences by PwO and CP. This is due in part to the subjectivity of pain experiences and, at times, an absence of structural pathology or diagnostic certainty within the narrow biomedical model of pain, more typically associated with acute pain conditions [87, 88, 89, 90]. However, employing reductionist frameworks can force unachievable objectivity on to complex health concepts [91]. Widening the gap between expectations and attainable outcomes undermines trust between HCPs and PwO and CP. This is demonstrated in our findings that showed participants using weight‐based criteria as a measure of perceived success or failure.

The evidence and guidelines regarding obesity care and management have shifted toward a more holistic focus [1, 2, 72, 92], yet there is a lack of clear and convincing evidence that the clinical paradigm shift has translated well to the patient perspective. This may, however, also be an artifact of the date range used for our searches, as many studies predate this important paradigm shift. It is crucial that HCPs focus on strategies to improve communication with patients and avoid inadvertently perpetuating the stigmatization of PwO and CP. Focusing on strategies aimed at improving empathy from HCPs (training, simulation, reflective practices, and improved care capacity and pathways that facilitate more time for consultations and nuanced discussion) can help mitigate care‐related stigma experienced by patients, making interactions more productive for both patients and HCPs [45, 87, 93].

While this study sheds light on the complex interaction between pain and obesity, further research questions remain. Mechanisms linking obesity and pain are complex and underexplored, while obesity and CP both remain undertreated [1, 94, 95, 96]. Obesity is fundamentally a disease of excess or dysfunctional adiposity. The experiences of pain in PwO may differ for individuals with dysfunctional adiposity compared to those individuals who have a higher BMI, but no further health concerns associated with their weight. Future research could help distinguish between these cohorts. Furthermore, while there is a growing body of evidence highlighting the need to address both pain‐associated and weight‐based stigma in healthcare provision, findings in this review and others [1, 96] suggest that this has yet to translate more broadly to clinical practice. Future research could explore how to more successfully bridge this gap in care between the evidence base and the lived experience of PwO and CP. This would result in important clinical solutions to existing inequalities of care [5, 97].

## 6 Strengths and Limitations

Strengths of this QES include that this study was prospectively registered and protocolized. QES was undertaken using a comprehensive, systematic, and methodologically rigorous approach to synthesizing the available qualitative evidence on living with obesity and chronic pain. Input from two patient insight partners validated the research question and assisted in reporting identified themes in plain language.

There are also some potential limitations. We only included peer‐reviewed studies published as full reports in the English language. Searching alternative sources, such as gray literature, and including studies published in other languages may have provided additional and/or alternative insights. Eight of the nine included studies were conducted in Western English‐speaking countries and cultures, which may restrict the generalizability of our findings to other geographical regions. Additionally, four of the nine studies included some participants with BMI < 30 kg/m^2^ although the mean BMI of the included participants was ≥30 kg/m^2^. While the decision to include these studies was based on agreement within the QES team, it is possible that the views of some people with overweight rather than obesity may have been included. Finally, five of the studies included participants predominantly experiencing knee OA, which may reflect an overrepresentation of views from people living with knee OA rather than a wider range of chronic pain conditions.

## 7 Conclusions

This QES characterizes the experiences of people living with obesity and co‐occurring CP. It identifies complex interactions between obesity and pain and the burden that PwO and CP experience. Addressing the co‐occurring pain of obesity should be part of obesity care. Healthcare professionals, policymakers, and funders should consider the provision of services to address the needs of people living with both obesity and CP.

## 8 Reflexivity Statement and Public Patient Involvement

Reflexivity requires researchers to consider how their personal beliefs and experiences relate to the phenomenon being explored. These factors can impact study design, results, analysis, and outputs. The authors in this review comprised academic and/or clinical physiotherapists with clinical and research interests in pain and obesity (N. S. H., M. B. C., C. G. D., C. D., C. B., B. M. F., G. O'D., and K. M. S.). The authors have varying degrees of experience and expertise in conducting qualitative research or evidence syntheses.

Prior to commencing the screening and analysis, the two review authors responsible for running the searches, screening for eligibility, and then extracting and analyzing data (N. S. H. and M. B. C.) met to discuss and consider their baseline epistemological viewpoints, preconceptions, and beliefs surrounding the subjects of obesity and pain [98]. At each stage of the analysis, these two reviewers discussed their expectations regarding the findings with respect to their experiences and perspectives. This was done in an effort to mitigate internally held biases that might otherwise influence outcomes at each stage of the research [99, 100].

Two voluntary “*Patient Insight Partners”* (S. B. and F. F.) collaborated with and advised the research team at several stages during the QES, one from the Irish Coalition for People Living with Obesity (ICPO) and one with lived experience of attending an Obesity Center of Care at a hospital in Dublin, Ireland. Both provided valuable insights into the wider context of living with both pain and obesity, interpreted the data, contributed to the preparation of this manuscript, and are included as coauthors. Public patient involvement (PPI) has been reported using Guidance for Reporting Involvement of Patients and the Public 2 (GRIPP2) reporting checklist and can be found in Data S6 [101].

## Author Contributions

N.S.H. is the lead author and guarantor. N.S.H. and K.M.S. planned this study, and N.S.H., M.B.C., and K.M.S. led the drafting and revising of the manuscript. N.S.H., M.B.C., C.D., C.B., B.M.F., C.G.G., G.O'D., S.B., F.F., and K.M.S. contributed to the interpretation of the background evidence, drafting of the manuscript, and revisions. All authors agreed on the submitted version of the manuscript.

## Ethics Statement

No ethical approval was required for this review.

## Conflicts of Interest

The authors declare no conflicts of interest.

## Supporting information


**Data S1.** ENTREQ checklist.
**Data S2.** Full table of characteristics of the included studies.
**Data S3.** Descriptive themes generated during Stage 2 of thematic synthesis.
**Data S4.** GRADE CERQual assessment of confidence in study findings.
**Data S5.** Themes and direct participant quotes.
**Data S6.** GRIPP2 reporting checklist.

## Data Availability

We have endeavored to make our data FAIR, that is, findable, accessible, interoperable, and reusable [102], by making data generated because of this synthesis, such as generated codes and themes, available in the Supporting Information.
