# Supplementary material for: “It Is a Vicious Circle”: Experiences of People Living With Obesity and Chronic Pain: A Qualitative Evidence Synthesis (QES)
Source: Obes Rev. 2025 Jul 28;27(1):e70004. doi: 10.1111/obr.70004 (PMC12685494; doi:10.1111/obr.70004)
Supplement: Supplementary file 1 — Data S1. ENTREQ checklist. Data S2. Full table of characteristics of the included studies. Data S3. Descriptive themes generated during Stage 2 of thematic synthesis. Data S4. GRADE CERQual assessment of confidence in study findings. Data S5. Themes and direct participant quotes. Data S6. GRIPP2 reporting checklist. [file OBR-27-e70004-s001.pdf]

Supporting Information

Title: "*It is a vicious circle*": Experiences of People Living with Obesity and Chronic Pain: A Qualitative Evidence Synthesis (QES)

Running Title: Experiences of People Living with Obesity and Chronic Pain

Authors: Natasha S Hinwood[1,2,3], Maire-Brid Casey[4], Colin G. Dunlevy[5], Catherine Doody[1,2], Catherine Blake[1,2], Bróna M. Fullen[1,2], Gráinne O'Donoghue[1], Susie Birney[6], Fionnuala Fildes[7], Keith M. Smart[1,2]

Corresponding author: Natasha Hinwood
Address: UCD School of Public Health, Physiotherapy and Sport Science, University College Dublin, Dublin, Ireland
Email: natasha.hinwood@ucdconnect.ie
Phone: +353 1 716 6511

Full name, department, institution, city, and country of all co-authors.
[1]UCD School of Public Health, Physiotherapy and Sport Science, University College Dublin, Dublin, Ireland
[2]UCD Centre for Translational Pain Research, University College Dublin, Dublin, Ireland
[3]Physiotherapy Department, the Beacon Hospital, Dublin, Ireland
[4]TCD Discipline of Physiotherapy, School of Medicine, Trinity College Dublin, Dublin, Ireland
[5] Centre for Obesity Management, St. Columcille's Hospital, Dublin, Ireland
[6]Irish Coalition for People Living with Obesity (ICPO), Dublin, Ireland
[7]Independent Patient Insight Partner, St. Vincent's Private Hospital, Dublin, Ireland

Supporting Information:
Supporting Information 1 - ENTREQ checklist
Supporting Information 2 - Full table of Characteristics of Included Studies
Supporting Information 3 – Descriptive Themes Generated During Stage 2 of Thematic Synthesis
Supporting Information 4 – GRADE CERQual Assessment of Confidence in Study Findings
Supporting Information 5 – Themes and Direct Participant Quotes
Supporting Information 6 - GRIPP2 Reporting Checklist

Supporting Information 1:<br>Enhancing transparency in reporting the synthesis of qualitative research (ENTREQ) Checklist			
Item		Guide and description	Pages
1	Aim	State the research question the synthesis addresses.	Page 2 (abstract) and page 3 (end of discussion section). Also outlined in published protocol (Hinwood et al. 2024)
2	Synthesis methodology	Identify the synthesis methodology or theoretical framework which underpins the synthesis and describe the rationale for choice of methodology *(e.g. meta-ethnography, thematic synthesis, critical interpretive synthesis, grounded theory synthesis, realist synthesis, meta-aggregation, meta-study, framework synthesis).*	Page 3-4 (methods). Also outlined in published protocol (Hinwood et al. 2024)
3	Approach to searching	Indicate whether the search was pre-planned (*comprehensive search strategies to seek all available studies)* or iterative (*to seek all available concepts until they theoretical saturation is achieved)*.	Page 3 (methods). Also outlined in published protocol (Hinwood et al. 2024)
4	Inclusion criteria	Specify the inclusion/exclusion criteria *(e.g. in terms of population, language, year limits, type of publication, study type).*	Page 3-4 (methods, deviations from protocol, results, Figure 1). Also outlined in published protocol (Hinwood et al. 2024)
5	Data sources	Describe the information sources used (e.g. *electronic databases (MEDLINE, EMBASE, CINAHL, psycINFO, Econlit), grey literature databases (digital thesis, policy reports), relevant organisational websites, experts, information specialists, generic web searches (Google Scholar) hand searching, reference lists)* and when the searches conducted; provide the rationale for using the data sources.	Pages 3-4 (methods). Also outlined in published protocol (Hinwood et al. 2024)
6	Electronic Search strategy	Describe the literature search *(e.g. provide electronic search strategies with population terms, clinical or health topic terms, experiential or social phenomena related terms, filters for qualitative research, and search limits)*.	Pages 3-4 (methods). Also outlined in published protocol (Hinwood et al. 2024)
7	Study screening methods	Describe the process of study screening and sifting *(e.g. title, abstract and full text review, number of independent reviewers who screened studies).*	Pages 3-5 (methods, deviations from protocol, results). Also outlined in published protocol (Hinwood et al. 2024).
8	Study characteristics	Present the characteristics of the included studies *(e.g. year of publication, country, population, number of participants, data collection, methodology, analysis, research questions).*	Pages 5-11 (results, summary of included studies, Tables 2, 3 and 4).
9	Study selection results	Identify the number of studies screened and provide reasons for study exclusion *(e,g, for comprehensive searching, provide numbers of studies screened and reasons for exclusion indicated in a figure/flowchart; for iterative searching describe reasons for study exclusion and inclusion based on modifications to the research question and/or contribution to theory development).*	Pages 4-5 (Results and Figure 2)
10	Rationale for appraisal	Describe the rationale and approach used to appraise the included studies or selected findings *(e.g. assessment of conduct (validity and robustness), assessment of reporting (transparency), assessment of content and utility of the findings).*	Pages 3-5 (methodology, results, quality of included studies, Table 4). Also outlined in published protocol (Hinwood et al. 2024).
11	Appraisal items	State the tools, frameworks and criteria used to appraise the studies or selected findings (e.g. Existing tools: CASP, QARI, COREQ, Mays and Pope; reviewer developed tools; describe the domains assessed: research team, study design, data analysis and interpretations, reporting).	CASP and GRADE-CERQual. Pages 3-5 (methodology, results, quality of included studies, Table 4). Also outlined in published protocol (Hinwood et al. 2024).
12	Appraisal process	Indicate whether the appraisal was conducted independently by more than one reviewer and if consensus was required.	Pages 3-5 (methodology, results, quality of included studies, Table 4). Also outlined in published protocol (Hinwood et al. 2024).
13	Appraisal results	Present results of the quality assessment and indicate which articles, if any, were weighted/excluded based on the assessment and give the rationale.	Pages 5 and 11 (quality of included studies, Table 4).
14	Data extraction	Indicate which sections of the primary studies were analysed and how were the data extracted from the primary studies? *(e.g. all text under the headings "results /conclusions" were extracted electronically and entered into a computer software).*	Pages 3-5 (methodology, results). Also outlined in published protocol (Hinwood et al. 2024).
15	Software	State the computer software used, if any.	Pages 4-5 (deviations from protocol and results). Also outlined in published protocol (Hinwood et al. 2024).
16	Number of reviewers	Identify who was involved in coding and analysis.	Pages 3-5 (methodology, results). Also outlined in published protocol (Hinwood et al. 2024).
17	Coding	Describe the process for coding of data *(e.g. line by line coding to search for concepts).*	Pages 3 and 5 (methodology and results. Also outlined in protocol (Hinwood et al. 2024).
18	Study comparison	Describe how were comparisons made within and across studies *(e.g. subsequent studies were coded into pre-existing concepts, and new concepts were created when deemed necessary).*	Pages 3 and 5 (methodology and results. Also outlined in protocol (Hinwood et al. 2024).
19	Derivation of themes	Explain whether the process of deriving the themes or constructs was inductive or deductive.	Pages 3 and 5 (methodology and results. Also outlined in protocol (Hinwood et al. 2024).
20	Quotations	Provide quotations from the primary studies to illustrate themes/constructs and identify whether the quotations were participant quotations of the author's interpretation.	Pages 12-17 (Themes and subthemes) and also in supplementary information 5.
21	Synthesis output	Present rich, compelling and useful results that go beyond a summary of the primary studies (e.g. *new interpretation, models of evidence, conceptual models, analytical framework, development of a new theory or construct).*	Pages 12-19 (Themes and subthemes, discussion).

Supporting Information 2: Full table of Characteristics of Included Studies

This provides details and study characteristics for each of the studies included in the review.

Supporting Information 2: Full table of Characteristics of Included Studies						
Study (country) and Sample size	Study Aim	Study Background	Sociodemographic and BMI	Pain Description	Methods	Themes Identified / Findings
Janke and Kozak. 2012 (USA)<br><br>n=30	To identify perceptions of people with overweight / PwO and CMP regarding experiences with<br>(i) barriers and facilitators for treatment<br>(ii) engagement with health-promoting behaviours<br>(iii) treatment for weight and/or pain control.	Author Characteristics:<br>Not discussed<br>Funding: Funded by a VA Health Services Research & Development Project Grant (TPP 42-004).<br>Conflicts of Interest:<br>None declared<br>Ethical Approval:<br>Approved by the hospital review board, and all procedures in accordance with the ethical standards of this review board.	Age: 86.6% (26 of 30) age ≥50<br>Gender: n=24 (of 30) (80%) male<br>Ethnicity: 73.3% (22 of 30) white<br>Employment Status: Unemployed or disabled, not-retired 46.6% (14 of 30); Retired 43.3% (13 of 30)<br>BMI Range: ≥25<br>BMI Mean (SD): 36.8 (8.9)	Average pain intensity 5.6 (SD 1.9); average pain interference 3.6 (SD 2.1); weekly pain at an intensity ≥4 (0 = none, 10 = worst imaginable) during the prior 3 months prior; current diagnosis of a medical complaint associated with persistent pain (e.g., osteoarthritis)	Design: Either individual interviews or focus groups (<4) with semi-structured discussion.<br>Setting: Primary care clinics large Midwestern Veteran's Affairs (VA) hospital.<br>Sampling: Purposeful<br>Recruitment: Flyers posted in hospital common areas and waiting rooms, and direct referral from providers.<br>Inclusion: (i) BMI ≥25; (ii) weekly pain at an intensity >4 (0 = none, 10 = worst imaginable) during the prior 3 months; and (iii) current diagnosis of a medical complaint associated with persistent pain (e.g., osteoarthritis).<br>Exclusion: (i) Individuals <18 years of age, (ii) inpatients, and those with (iv) difficulty communicating in English, (v) active substance abuse or (vi) pain was exclusively cancer related.<br>Analysis: Constant comparative method.	(i) Role of depression;<br>(ii) Pain, shame, and hedonic hunger;<br>(iii) Emotional eating and pain;<br>(iv) Altered dietary choices;<br>(v) Reduced engagement in and low self-efficacy for physical activity
Bunzil, *et al.* 2019 (Australia)<br><br>n=27	To investigate the patient-related factors that affect treatment decision, such as<br>(i) beliefs/attitudes toward knee OA<br>(ii) beliefs/attitudes toward treatment<br>(iii) health system-related factors (access, referral pathways).	Author Characteristics:<br>Not discussed<br>Funding: Funding unclear - one of the authors (MD) holds a National Health & Medical Research Council (NHMRC) Career Development Fellowship (1122526) and one or more of the authors (MD, PC, JG) hold an NHMRC Center for Research Excellence Grant in Total Joint Replacement (1116325).<br>Conflicts of Interest:<br>All ICMJE Conflict of Interest Forms available upon request.<br>Ethical Approval:<br>Each author certified that his or her institution approved the human protocol for this study.	Age: mean 68 years; range 50-80+<br>Gender: n=13 (of 27) (48%) female<br>Ethnicity: Not reported<br>Employment Status: Not reported<br>BMI Range: 19 - 30+; Overweight (11), PwO (16)<br>BMI Mean (SD):<br>33 (Not reported)	End stage knee OA, awaiting TKR	Design: Individual interviews (in-person and phone) using an interview guide.<br>Setting: Orthopaedic clinic of a large tertiary hospital in a metropolitan region of Australia.<br>Sampling: Not reported<br>Recruitment: Patients on the waiting list for TKA attending the orthopaedic preadmission clinic were approached by a research assistant and invited to participate.<br>Inclusion: People older than 18 years, spoke English, had a diagnosis of knee OA, and had consented to undergo primary TKA. Nil BMI restrictions.<br>Exclusion: Patients were ineligible if they needed an interpreter or were unable to provide independent informed consent for TKA because of cognitive impairment.<br>Analysis: Analysis using the Five Step Framework approach, using coding informed by health belief model (common Sense Model).	(i) Identity belief: *'Knee OA is bone on bone'*;<br>(ii) Causal belief: *'OA is due to excessive loading through the knee'*;<br>(iii) Consequence beliefs: *'Fear of falling and damaging the joint'*;<br>(iv) Timeline beliefs: *'OA as a downward trajectory'; 'The urgency to do something'; 'Arriving at the end of the road'*;<br>(v) Treatment beliefs: *'The weight loss dilemma'; 'Physiotherapy can't help bone on bone'; 'Replacing the cartilage'; 'A mechanical problem requires a mechanical fix'*

Cooper, *et al*. 2018 (UK)<br><br>n=18	To gain insight into the lived experience of adults with overweight/obesity and CMP.	Author Characteristics:<br>Not discussed<br><br>Funding: Not stated<br><br>Conflicts of Interest:<br>None declared<br><br>Ethical Approval:<br>Yes - ethical approval to conduct the study was obtained from Teesside University School of Health and Social Care Research Governance Ethical Committee.	Age: 29 to 71; mean 53.5 (SD 11)<br><br>Gender: n=16 (of 18) (89%) female<br><br>Ethnicity: Not reported<br><br>Employment Status: Not reported<br><br>BMI Range: 25 - 45.9; Overweight (6), PwO (12)<br><br>BMI Mean:<br>33.27 (5.94)	Mean pain score was 4.6 (out of 10) for intensity. Inclusion criteria was self-reported persistent musculoskeletal pain ≥4/10 (0 = no pain, 10 = worst pain) any site for >3 months. Participants reported pain in various sites, but particularly in their knees and lower back, with several reporting pain in multiple sites. Most of the participants reported that pain affected their enjoyment of life and ability to sleep. Several felt it affected their relationships with others.	Design: Face-to-face in-depth, semi-structured interviews.<br>Setting: Participants recruited from regional branches of a commercial weight loss service.<br><br>Sampling: Purposeful<br><br>Recruitment: Direct recruiting by research team, following a presentation, with the opportunity to speak to the researcher privately.<br>Inclusion: People (i) currently attending the commercial weight loss service; (ii) aged≥18 years, (iii) BMI≥25; (iv) self-reported persistent musculoskeletal pain≥4/10 any site for>3 months and occurring most days.<br><br>Exclusion: Pregnant or breastfeeding women and those who did not speak English were excluded.<br><br>Analysis: Interpretive phenomenological analysis.	(i) Pain as a motivator and barrier to weight loss;<br>(ii) Fear of weight causing more damage;<br>(iii) Activity, at least certain types of activity, is positive
Craft, *et al*. 2015 (USA)<br><br>n=15	To find out from women with FM<br>(i) their needs and preferences for, and<br>(ii) barriers to participation in weight management programs.	Author Characteristics:<br>Not discussed<br><br>Funding:<br>The Mayo CCaTS grant number UL1TR000135.<br><br>Conflicts of Interest:<br>Not discussed<br><br>Ethical Approval: Approved by the Mayo Clinic Institutional Review Board and all patients provided written informed consent.	Age: mean 50.8 (SD 6.3)<br><br>Gender: N=15 (100%) female<br><br>Ethnicity:<br>Race, white, N (%) 14 (93.3)<br>Employment Status: Employed full time 8(53.3); part-time 3(20.0) ; unemployed 3(20.0) ; homemaker 1(0.1)<br><br>BMI Range:<br>≥30<br><br>BMI Mean (SD):<br>37.88 (4.87)	Diagnosis of fibromyalgia (FM). The mean total score on the Fibromyalgia Impact Questionnaire (FIQR) was 56.5 12.5, indicating a high-moderate effect of symptoms overall. Individual mean scores on the Patient Health Questionnaire (PHQ-9) ranged from 2 to 19 (12.9 - 4.9), indicating moderate depressive symptomology.	Design: Focus group discussion<br><br>Setting: Community dwelling, patients who had previously attended a fibromyalgia treatment program (FTP) within the prior year.<br><br>Sampling: Not reported<br><br>Recruitment: Invitation letter to participate, sent by post.<br><br>Inclusion: (i) attendance in the fibromyalgia treatment program (FTP) within the prior year, (ii) BMI of ≥ 30, (iii) 30–60 years of age, (iv) diagnosis of FM based upon either or both of the American College of Rheumatology 19901 and 201014 criteria, (v) residing in Minnesota, (vi) agreed to the Minnesota Research Authorization, allowing contact for participation in studies, and (vii) female gender<br>Exclusion: Not discussed<br><br>Analysis: Thematic analysis.	(i) There are complex connections between fibromyalgia, weight, and exercise;<br>(ii) Healthy eating can be burdensome and time consuming for women with fibromyalgia;<br>(iii) Emotions play a key role in these connections, and women often feel misunderstood;<br>(iv) There is a need for specialized weight management programs specifically for women with fibromyalgia.
Godziuk, *et al*. 2022 (Canada)<br><br>n=20	To incorporate perspectives of people with OA and BMI>35 in the design and delivery of a multimodal intervention intended to prevent muscle loss.	Author Characteristics:<br>Not discussed<br>Funding: Not supported by any specific funding or grant.<br>Conflicts of Interest: KG and MF have no conflicts of interest to declare. Carla M.	Age: mean age 54.7 years (SD 9.7); age range 41- 68<br>Gender: N=20 (100%) female<br>Ethnicity:<br>Not reported<br>Employment Status:<br>Work full-time or part-time 15	Self-reported knee OA, predominantly reported bilateral knee OA (75%), symptom onset ≥5 years prior (85%); self-reported severity: mild 4 (20),	Design: After reading about a 12-week intervention, participants answered anonymous open-ended electronic survey questions, with the option of a one-on-one semi-structured interview afterwards.<br>Setting: Anonymous online members of the public.<br><br>Sampling: Purposeful and voluntary sampling.<br><br>Recruitment: Anonymous electronic survey, distributed on the Obesity Canada (OC) website and social media.	(i). Emphasise 'health gains' over 'weight loss';<br>(ii). Support and language matter;<br>(iii). Include options for customisation.

		Prado has received paid consultancy from several activities unrelated to this work.<br><br>Ethical Approval: Prior approval from the University of Alberta Health Ethics Review Board	(75); On disability leave 2 (10); Retired, laid-off, unreported 3 (15)<br><br>BMI Range: ≥35<br><br>BMI Mean (SD):<br>Not reported	moderate 9 (45), severe 7 (35)	Inclusion: (i) Participants ≥40 years old; (ii) had a BMI≥35 kg/m2; (iii) and had been told by a doctor that they have arthritis or OA in one or both knees. All self-reported.<br>Exclusion: Not discussed.<br>Analysis: Framework analysis, with thematic analysis.	
Janke, *et al*. 2015 (USA)<br><br>n=30	To understand patients'<br>(i) preferences and<br>(ii) dissatisfaction<br>with pain and obesity care in a primary care setting.	Author Characteristics:<br>Not discussed<br>Funding: Funded by a VA Health Services Research & Development Project Grant (TPP 42-004).<br><br>Conflicts of Interest:<br>None declared<br><br>Ethical Approval: The local Institutional Review Board approved the study.	Age: 86.6% (26 of 30) age ≥50<br>Gender: n=24 (of 30) (80%) male<br>Ethnicity: 73.3% (22 of 30) white<br>Employment Status: Unemployed or disabled, not-retired 46.6% (14 of 30); Retired 43.3% (13 of 30)<br>BMI Range:<br>≥25<br><br>BMI Mean (SD):<br>36.8 (8.9)	Average pain intensity 5.6 (SD 1.9); average pain interference 3.6 (SD 2.1); weekly pain at an intensity ≥4 (0 = none, 10 = worst imaginable) during the prior 3 months prior; current diagnosis of a medical complaint associated with persistent pain (e.g., osteoarthritis)	Design: Either individual interviews or focus groups (<4) with semi-structured discussion.<br>Setting: Primary care clinics large Midwestern Veteran's Affairs (VA) hospital.<br>Sampling: Purposeful<br>Recruitment: Flyers posted in hospital common areas and waiting rooms, and direct referral from providers.<br><br>Inclusion: (i) BMI ≥25; (ii) weekly pain at an intensity >4 (0 = none, 10 = worst imaginable) during the prior 3 months; and (iii) current diagnosis of a medical complaint associated with persistent pain (e.g., osteoarthritis).<br>Exclusion: (i) Individuals <18 years of age, (ii) inpatients, and those with (iv) difficulty communicating in English, (v) active substance abuse or (vi) pain was exclusively cancer related.<br>Analysis: Analysis used the constant comparative method.	(i) Need for information tailored to comorbidity;<br>(ii) Frustration with available treatment approaches and desire for motivation enhancement;<br>(iii) Provider initiated concern and communication
O'Neill and Worboys. 2011 (UK)<br><br>n=1	To look at the patient journey and the challenges of a patient attending the service. The patient was a person living with obesity and lymphoedema.	Author Characteristics:<br>FW is Lead Nurse, Tissue Viability Team and CO'N is a Lymphoedema Specialist.<br>Funding:<br>Not discussed<br>Conflicts of Interest:<br>Not discussed<br>Ethical Approval:<br>Not discussed	Age: 66 years old<br>Gender: N=1 male<br>Ethnicity: Not reported<br>Employment Status: Not reported<br><br>BMI Range: Not reported<br><br>BMI Mean (SD):<br>BMI > 30	Pain associated with 10-year history of lymphoedema	Design: A short interview with the patient on his experience as a patient living with lymphoedema.<br>Setting: The patient presented as an outpatient clinic at the East London wound healing service.<br>Sampling: Not discussed.<br><br>Recruitment: Not discussed.<br><br>Inclusion: Not discussed.<br><br>Exclusion: Not discussed.<br>Analysis: Nil structured analysis described.	That the patient's management was largely autonomously driven which underscored the need for investment in related services.
Toye, *et al*. 2018 (UK)<br><br>n=6	To explore barriers to weight loss in a group of older men with osteoarthritis.	Author Characteristics: A female anthropologist, a male physiotherapist (JR) and a female physiotherapist (KB).<br><br>Funding: Not discussed<br><br>Conflicts of Interest:<br>None declared<br>Ethical Approval: Research was given favorable review by the local national research ethics committee (NREC) (09/h0606/87)	Age: aged 59 to 76 years<br><br>Gender: N=6 (100%) male<br><br>Ethnicity: All white (British)<br>Employment Status:<br>Employed = 4; retired = 2<br>BMI Range:<br>31 - 38<br>BMI Mean (SD):<br>33 (not reported)	Knee OA, awaiting knee joint replacement	Design: Two sets of semi-structured interviews with men who had undergone total knee replacement (TKR). One prior to joint replacement surgery and one 12-months post-op.<br>Setting: Participants interviewed at home (n=5), or at the hospital (n=1). Two patients were joined by their wives during the interviews.<br>Sampling: Not discussed.<br><br>Recruitment: Not specifically described. Men who underwent surgery (TKR).<br><br>Inclusion: Men who underwent surgery (TKR). Not discussed explicitly or in further detail.<br><br>Exclusion: Not discussed.<br><br>Analysis: Analysis using a constructivist grounded theory approach.	There were tensions in men's body talk that might influence their healthcare decisions: *'I am big and healthy and don't need to lose weight'*, and yet *'being this size can't be that good for me'*. Men discussed reasons that they might put on weight which might be potential barriers to weight loss.

Storm, *et al*. 2023 (Sweden)<br><br>n=16	To explore patient perspectives on how chronic pain and obesity influenced<br>(i) each other; (ii) ability to make lifestyle changes.	Author Characteristics:<br>Researchers with different clinical professions (medical student, physician, occupational therapists, and psychologist) and with various research experience.<br>Funding: Open access funding provided by Linköping University, with grants from the Fibromyalgia Association of Sweden (2022) and the County Council of Östergötland (SC-2021).<br>Conflicts of Interest:<br>None declared<br><br>Ethical Approval:<br>Study was approved by the Swedish Ethical Review Authority (Dnr: 2021/028−11).	Age: mean 43.8 (SD 10.2); range 28–63<br>Gender: n=11 (69%) female, n=5 (31%) male<br><br>Ethnicity: All Swedish<br><br>Employment Status:<br>Employed = 11<br>BMI Range: 30–43<br><br>BMI Mean (SD):<br>35.7 (4.4)	Pain intensity (scale 0–10), Mean ± SD 5.5 ± 1.7 2.5–8; Pain duration, years, Median (Q1-Q3) 8 (4.4–20.8) ; Pain diagnosis: Fibromyalgia/widespread pain (5), Lower back pain (4), Hypermobility syndromes (4), Joint pain (2), Myalgia (not specified) (1)	Design: Semi-structured interviews, either by phone or in-person at the pain clinics, using an interview guide.<br>Setting: Previous patients of an 8-week Interdisciplinary Pain Rehabilitation Program in two specialist pain rehabilitation clinics in Sweden.<br><br>Sampling: Purposeful<br><br>Recruitment: Patients contacted consecutively from the Swedish Quality Registry for Pain Rehabilitation (SQRP) and invited to participate.<br>Inclusion: Clinic patients with (i) chronic pain and (ii) obesity (Body Mass Index (BMI >30 kg/m), (iii) who had completed an IPRP between 2019 and 2021.<br>Exclusion: No discussed.<br><br>Analysis: Latent content analysis.	i) Lifestyle changes are burdensome with a body broken by both pain and obesity; (ii) Pain disturbing days and nights worsens weight control; (iii) Pain-related stress makes lifestyle changes harder; (iv) A body affected by pain and obesity, intertwined with negative emotions (v) The overlooked impact of obesity on chronic pain.
Lawford, *et al*. 2023 (Australia)<br><br>n=20	To explore experiences of people with knee OA who are aiming to maintain weight loss following a multicomponent remotely delivered, clinician-supported weight loss programme.	Author Characteristics:<br>Not discussed<br>Funding:<br>Funded by Medibank and the Medibank Better Health Foundation Research Fund & National Health and Medical Research Council (NHMRC) Centre of Research Excellence (APP1079078). Funders had some involvement in the RCT design and recruitment.<br><br>Conflicts of Interest:<br>None declared<br><br>Ethical Approval:<br>all procedures involving research study participants were approved by the University of Melbourne Human Research Ethics Committee (#12849).	Age: mean 65 years (SD 9.0)<br>Gender:<br>n=10 (50%) female,<br>n=10 (50%) male<br>Ethnicity:<br>Not reported<br>Employment Status:<br>Not reported<br>BMI Range:<br>28.1 - 37.9<br><br>BMI Mean (SD):<br>32.7 (2.9)	Knee pain at baseline mean 5.6 (SD 1.4); knee pain at 6 months post-intervention 1.6 (SD 1.3); history of knee pain on most days for at least 3 months and met OA criteria	Design: Nested qualitative study. Semi-structured interviews conducted over the telephone with participants who had been randomised to the weight loss arm of an RCT and had successfully lost weight.<br>Setting: Community dwelling adults with private health insurance from across Australia.<br><br>Sampling: Not reported<br><br>Recruitment: Participants were invited 1–3 weeks of having completed their final 12-month questionnaire for the RCT. Recruitment for RCT was done through the private health insurance company by targeted emails.<br>Inclusion: Previously participated in the RCT. Eligibility for the RCT: (i) held private health insurance with a specific insurer that included cover for arthroplasty surgery; (ii) met the National Institute for Health and Care Excellence OA clinical criteria (aged 45 years, activity-related joint pain, morning stiffness 30min);10 (ii) had average knee pain 4 on 11-point numeric rating scale (0=no pain, 10=worst pain possible) in the past week; (iv) had a history of knee pain on most days for at least 3 months; (v) were aged <81 years and (vi) had a BMI 28kg/m2 and <41kg/m2.<br>Exclusion: Same as the exclusion criteria for the RCT, including recent knee surgery (6 months) or due surgery, unable to participate in VLCD. Full eligibility criteria reported in the trial protocol [59].<br><br>Analysis: Inductive thematic analysis.	(i) Successfully maintained weight loss;<br>(ii) Empowering self-management of weight (understanding importance of exercise and physical activity, increased knowledge about food and nutrition, resources from programme still useful, knee pain as a motivator, have confidence in ability to self-regulate weight;<br>(iii) Challenges keeping on track

Supporting Information 3: Descriptive Themes Generated During Stage 2 of Thematic Synthesis

This an outline of the initial 36 descriptive themes generated in Step 2 of the synthesis process, from the 250 unique codes developed from the line-by-line coding of the included data. The 36 descriptive themes were then further amalgamated into 18 themes as listed below.

**Supporting Information 3**: Descriptive Themes Generated During Stage 2 of Thematic Synthesis
Descriptive themes (Stage 2, Round 1)
1. PwO and CMP have needs unique compared to that of the needs of general population, with a sense of being not well understood by society at large<br>2. Unique populations (men / gendered beliefs, FM) having specific beliefs (e.g. larger bodies perceived as healthy) with unique needs<br>3. Primarily biomechanical ('wear and tear') understanding of pain as a foundation for a cluster of related beliefs e.g. kinesiophobia, weight causing pain, that weight needs to decrease to improve pain (but also that pain needs to improve to lose weight); fear that more weight is causing more damage; that more movement is causing more damage; body is failing with age; only mechanical solutions (surgery) will 'fix' problems and doubt about personal ability to influence situation / sense of futility in attempting management via alternative solutions<br>4. Recognition of exercise benefits<br>5. Disbelief pain and weight are related and 'mind over matter' belief to weight loss<br>6. Decreased physical activity and increased sedentary activity contributing to increased obesity, with fatigue being a large barrier to increased physical activity<br>7. Decreased physical activity due to pain, low motivation and self-efficacy for physical activity<br>8. Weight loss allowing more physical activity, but more physical activity not accounting for more weight loss (unidirectional benefits)<br>9. Motivation to engage with exercise despite multiple barriers and multiple failed attempts due to exercise exacerbating pain<br>10. Need for 'safe' and achievable (physically and financially // in terms of all the barriers) exercise due to fear of further damage and fatigue<br>11. Evidence for positive self-efficacy - Motivated for exercise and self-management in achievable and controllable manner (e.g. self-soothing with food or by reducing activity to manage fatigue, constant trying despite failing)<br>12. Physical activity causing pain, undermining motivation or self-efficacy for the same<br>13. Perceived progress improving self-efficacy<br>14. Self-blame, sense of personal failure (exacerbated by mind over matter) and embarrassment regarding body size affecting interactions with external environment (exercising in public, social, sensitivity discussing topic) leading to a sense of loneliness and isolation<br>15. Mood amplification of other factors (good mood can amplify in positive, low mood can amplify in negative), particularly important in the context of low mood. Low mood and emotional distress exacerbates and amplifies pain and complicates efforts to cope with chronic pain and co-occurring obesity in a cyclical pattern.<br>16. Altered sense of self-image and identity // sense of comparison with pre- and post- obesity and pain<br>17. Negative psychological factors (depression especially, shame, fear, loneliness, frustration) complicating treatment for comorbid pain and obesity<br>18. Chronic sense of body fatigue and failure / fighting own body and lack of control, altering self-image and self-efficacy-fatigue for lifestyle changes or PA<br>19. Frustration with and sense of inadequacy of systemic support from HCP regarding continuity of care, creativity of treatment approaches, language and communication, empathy and education with respect to overall experience of living with concomitant pain and obesity, which overall undermine trust in system

20. Capricious relationship with HCP - at times very challenging and communication at times lacking empathy, not patient centred (e.g. when centred around HCP goals vs patient's goals) and demonstrative of bias or stigmatising beliefs around blame for weight and pain
21. Recognition for need for support for lifestyle change & behaviour change guidance from HCP and peers
22. Recognition for the diverse set of needs overall, lack of homogeneity and need for creative personalisation & individualisation of holistic and multifactorial treatment options with synergistic gains (gender, disease background & PMH, ability)
23. Need for sustainable treatment options and continuity of care with need for long-term support from both HCP and inclusive community & peer support
24. Need for further education and help to build confidence to address barriers to engaging with treatment options (such as fatigue) and focus on facilitating factors
25. Whole-person approach: focus on health gain versus weight loss to help sustain engagement and lifestyle changes; and focus on small gains to amplify
26. Recognition of the challenges regarding communication with HCP
27. Stigma and external blame experienced in multiple settings (home, gym or exercise spaces, health care interactions)
28. The (long term) experience of living with chronic pain and obesity has a negative psychosocial impact (combined burden of comorbid diseases) and alters engagement with external world, including around healthful behaviours through self-efficacy-fatigue and downregulation (?) of motivation to engage with healthy behaviours (food, social connection) and exercise.
29. Pain is both a motivator for and a barrier to weight loss for PwO and CMP. Pain is a barrier in terms of affecting function, sleep.
30. Altered food-related responses (increased frequency and volume, with poorer quality and difficulty managing cravings) as a response to both pain and distress (hedonic hunger as a response to emotional distress & physical pain, boredom eating,) - at times described by 'addiction to eating' (unsure if meets clinical criteria for same - but interesting term intentionally used)
31. Barriers to 'healthy' eating including time, fatigue, education (lifelong patterns with roots in familial understanding of food, and nutritional education), access to good quality food, economic barriers
32. Difficulty with freedom vs restriction balance (strict dieting vs eating everything, difficulty with moderation, difficulty with dieting)
33. Family dynamics have strong influence on experience of PwO and CMP
34. Extrinsic barriers, such as access to care, obesogenic environment, sedentary Western lifestyle, food industry factors, obesogenic medications, ability to purchase a bike and equipment suitable for PwO
35. Multiple barriers to weight loss
36. Obesity is multifactorial, with many reasons for weight gain (including life events) and a lot of barriers to weight loss despite this being considered important for PwO for both aesthetic and functional purposes

New Descriptive Themes (Stage 2, Round 2)

1. Unique populations (men / gendered beliefs, FM) having specific beliefs (e.g. larger bodies perceived as healthy) with unique needs
2. Primarily biomechanical ('wear and tear') understanding of pain as a foundation for a cluster of related beliefs e.g. kinesiophobia, weight causing pain, that weight needs to decrease to improve pain (but also that pain needs to improve to lose weight); fear that more weight is causing more damage; fear that more movement is causing more damage; body is failing with age; only mechanical solutions (surgery) will 'fix' problems and doubt about personal ability to influence situation / sense of futility in attempting management via alternative solutions
3. Recognition of exercise benefits
4, Decreased physical activity and increased sedentary activity contributing to increased obesity, with fatigue being a large barrier to increased physical activity, Pain is both a motivator for and a barrier to weight loss for PwO and CMP. Pain is a barrier in terms of affecting function, sleep; Decreased physical activity due to pain, low motivation and self-efficacy for physical activity

5. All things self-efficacy (positive and negative; including evidence for positive self-efficacy - Motivated for exercise and self-management in achievable and controllable manner (e.g. self-soothing with food or by reducing activity to manage fatigue, constant trying despite failing), Physical activity causing pain, undermining motivation or self-efficacy for the same, and perceived progress improving self-efficacy<br>6. Self-blame, sense of personal failure (exacerbated by mind over matter) and embarrassment regarding body size affecting interactions with external environment (exercising in public, social, sensitivity discussing topic) leading to a sense of loneliness and isolation, Altered sense of self-image and identity // sense of comparison with pre- and post- obesity and pain, Chronic sense of body fatigue and failure / fighting own body and lack of control, altering self-image and self-efficacy-fatigue for lifestyle changes or PA<br>7. Mood amplification of other factors (good mood can amplify in positive, low mood can amplify in negative), particularly important in the context of low mood. Low mood and emotional distress exacerbates and amplifies pain and complicates efforts to cope with chronic pain and co-occurring obesity in a cyclical pattern. Negative psychological factors (depression especially, shame, fear, loneliness, frustration) complicating treatment for comorbid pain and obesity.<br>8. Negative life events & trauma predisposing to obesity with stress from Rx<br>9. Frustration with and sense of inadequacy of systemic support from HCP regarding continuity of care, creativity of treatment approaches, language and communication, empathy and education with respect to overall experience of living with concomitant pain and obesity, which overall undermine trust in system, while also recognising benefits and need for support from HCP<br>10. Whole-person approach: focus on health gain versus weight loss to help sustain engagement and lifestyle changes; and focus on small gains to amplify, need for 'safety' in terms of exercises and Rx (think of barriers, Cooper at al argue BMI cut off)<br>11. Stigma (all) and external blame experienced in multiple settings (home, gym or exercise spaces, health care interactions), including 'mind over matter' belief regarding weight loss, A sense of being not well understood by society at large<br>12. The (long term) experience of living with chronic pain and obesity has a negative psychosocial impact (combined burden of comorbid diseases) and alters engagement with external world, including around healthful behaviours through self-efficacy-fatigue and downregulation (?) of motivation to engage with healthy behaviours (food, social connection) and exercise.<br>13. People's understanding of their own pain and obesity and the pain and the relationship between those 2 concepts<br>14. Altered food-related responses (all) (increased frequency and volume, with poorer quality and difficulty managing cravings) as a response to both pain and distress (hedonic hunger as a response to emotional distress & physical pain, boredom eating,), with difficulty with restriction - at times described by 'addiction to eating' (unsure if meets clinical criteria for same - but interesting term intentionally used)<br>15. Barriers to 'healthy' eating including time, fatigue, education (lifelong patterns with roots in familial understanding of food, and nutritional education), access to good quality food, economic barriers<br>16. Family dynamics have strong influence on experience of PwO and CMP<br>17. Extrinsic barriers, such as access to care, obesogenic environment, sedentary Western lifestyle, food industry factors, obesogenic medications, ability to purchase a bike and equipment suitable for PwO<br>18. Obesity is multifactorial, with many reasons for weight gain (including life events) and a lot of barriers to weight loss despite this being considered important for PwO for both aesthetic and functional purposes

Supporting Information 4: <u>GRADE CERQual Assessment of Confidence in Study Findings</u>

This provides an assessment of the confidence found in each of the review findings, using the GRADE CERQual Assessment tool. Each finding is appraised separately to give an overall assessment of confidence in that review finding, based on the criteria of i. methodological limitations; ii. Coherence; iii. adequacy; and iv. relevance.

Supporting Information 4: GRADE CERQual Assessment of Confidence in Study Findings

Finding		Methodological Limitations	Coherence	Adequacy	Relevance	Overall assessment of confidence	Explanation of GRADE-CERQual assessment	References
1	Theme 1: A Predominant Bio-Mechanical Understanding of Pain<br><br>PwO and CP making sense of pain and reaching a predominantly biomechanical understanding of pain with a cluster of related beliefs (kinesiophobia; lack of confidence in their own body to perform basic physical activities, such as walking; weight-gain as the cause of pain; that weight needed to decrease to improve pain; that tissue or bodily degradation was inevitable due to age; only mechanical solutions and external factors (i.e. surgery) would resolve the pain; futility regarding ability to self-manage pain and weight).	Moderate concerns regarding methodological limitations because of the 8 studies that contributed - 1 had nil concerns (50 - Cooper), 6 had minor (48 - Janke 2012, 49 - Bunzil 2019, 52 - Godziuk 2022, 56 - Storm and 57 - Lawford), 1 (55 - Toye) had major concerns re methodology / clarity. Most concerns were re: relationship researcher and participant.	Nil / very minor concerns regarding coherence. Some slightly conflicting data present, but heterogeneity likely due to population heterogeneity and presented in findings.	Nil / very minor concerns regarding adequacy. Mostly very rich data with thick contextual description.	Minor concerns regarding relevance as all studies came from five high-income countries. Therefore, global relevance is limited/uncertain.	High confidence	High confidence due to the consistency across papers, with rich descriptive data to support the same.	Janke 2012; Bunzil 2019; Cooper 2018; Godziuk 2022; Janke 2015; Toye 2018; Storm 2023; Lawford 2024;
1.1	Subtheme 1.1: Healthcare Professionals' Language Shapes Understanding<br><br>The role of communication with Health Care Professionals (HCPs) within the search for understanding. Potentially inappropriate use of language from HCPs led to misinterpretation, inadvertently reinforcing biomechanical and fear-based beliefs.	Moderate concerns regarding methodological limitations because of the 6 studies that contributed - 1 had nil concerns, 4 had minor, 1 (54 - ONeill) had major concerns regarding methodology / clarity. Most other concerns regarding relationship between researcher and participant.	Nil / very minor concerns regarding coherence.	Minor concerns regarding adequacy as only six of the ten studies contributed to this finding and one of the studies (O'Neill) provided very thin data. Other studies gave rich data with thick contextual description.	Minor concerns regarding relevance as all studies came from three high-income countries. Therefore, global relevance is limited/uncertain.	Moderate confidence	Moderate confidence due to the absence of supportive data of some papers. Moderate concerns regarding the methodological rigour and adequacy of some of the papers that contributed to this finding.	Janke 2012; Cooper 2018; Craft 2015; Godziuk 2022; Janke 2015; O'Neill 2011;
1.2	Subtheme 1.2: Seeking 'Safe' Exercise: Navigating Fear of Pain and Damage<br><br>Threat perception and the importance of '*safety*'. Participants expressed a need for '*safety*' during exercise in terms of type and quantity, but also in terms of psychological support managing the perceived threat of movement, regulating mood and engaging with exercise, from sources who understood their comorbidities and complexity.	Moderate concerns regarding methodological limitations. Of the 7 studies that contributed - 1 had nil concerns, 5 had minor, and 1 (O Neill) had major concerns regarding methodological clarity. Most other concerns related to relationship between researcher and participant.	Nil / very minor concerns regarding coherence.	Nil / very minor concerns. Well supported by more than half of the included studies and richest data came from most methodologically robust studies. Some transformation and theoretical interpretation.	Minor concerns regarding relevance as all studies came from four high-income countries. Therefore, global relevance is limited/uncertain.	High confidence	High confidence due to the consistency across most papers, with rich data to support the same. Some concerns re: methodology, but not considered impactful.	Janke 2012; Bunzil 2019; Cooper 2018; Craft 2015; Godziuk 2022; O'Neill 2011; Lawford 2024;

	Finding	Methodological Limitations	Coherence	Adequacy	Relevance	Overall assessment of confidence	Explanation of GRADE-CERQual assessment	References
2	Theme 2: Catch 22: Vicious Cycle of Pain and Obesity<br><br>Catch 22: The cyclic duality of living with obesity and pain. Frustration stemming from the relentlessness and cyclical nature of trying to manage both chronic pain and obesity simultaneously. The role of poor mental well-being in amplifying this cycle.	Moderate concerns regarding methodological limitations. Of the 10 studies that contributed - 1 had nil concerns, 7 had minor, 2 (Toye and O Neill) had major concerns regarding methodological clarity. Most other concerns were related to relationship between researcher and participant.	Minor concerns regarding coherence. Some slightly conflicting data within the studies (Storm 2023, Godziuk 2022), but this was also discussed by both study authors and reflected in the synthesised theme.	Nil / very minor concerns regarding adequacy. Well supported by half of the studies with rich data. More thin data in the remaining half of the studies but supported throughout all studies in some way. Some transformation and theoretical interpretation.	Minor concerns regarding relevance as all studies came from five high-income countries. Therefore, global relevance is limited/uncertain.	Moderate confidence	Moderate confidence overall. There is some conflict within the data, but this may be representative of a heterogeneous population and is represented in the theme.	Janke 2012; Bunzil 2019; Cooper 2018; Craft 2015; Godziuk 2022; Janke 2015; O'Neill 2011; Toye 2018; Storm 2023; Lawford 2024;
2.1	Subtheme 2.1: Pain and Obesity: Both Motivators and Barriers<br><br>Participants also commonly viewed pain as both a motivator and a barrier. However, many participants expressed frustration and perceived poor success with trying to manage both pain and obesity through mechanisms focused mainly on weight-loss, such as dieting or engaging with exercise.	Moderate concerns regarding methodological limitations. Of the 10 studies that contributed - 1 had nil concerns [50 – cooper], 7 had minor, 2 (55 - Toye and 54 – O'Neill) had major concerns regarding methodological clarity). Most other concerns were related to relationship between researcher and participant.	Minor concerns regarding coherence. Some slightly conflicting data within the studies (Storm 2023, Godziuk 2022), but this was also discussed by both study authors and reflected in the synthesised theme.	Nil / very minor concerns regarding adequacy. Well supported by half of the studies with rich data. More thin data in the remaining half of the studies but supported throughout all studies in some way. Some transformation and theoretical interpretation.	Minor concerns regarding relevance as all studies came from five high-income countries. Therefore, global relevance is limited/uncertain.	Moderate confidence	Moderate confidence overall. There is some conflict within the data, but this may be representative of a heterogeneous population and is represented in the theme.	Janke 2012; Bunzil 2019; Cooper 2018; Craft 2015; Godziuk 2022; Janke 2015; O'Neill 2011; Toye 2018; Storm 2023; Lawford 2024;
2.2	Subtheme 2.2: The Impact of Negative Emotions on Pain<br><br>Thoughts and feelings associated with pain, highlighting the central role of emotions for PwO and CP. How emotions impact experiences of CP pain and obesity - predominantly related to the role of low mood, frustration, and depression - commonly described as a significant barrier to improving experiences due to the amplifying effect of low mood on pain.	Moderate concerns regarding methodological limitations. Of the 10 studies that contributed - 1 had nil concerns [50 – cooper], 7 had minor, 2 (55 - Toye and 54 - O'Neill) had major concerns regarding methodological clarity. Most other concerns were related to relationship between researcher and participant.	Nil / very minor concerns regarding coherence.	Nil / minor concerns. Mixture of thinner and thicker data, but mostly rich and descriptive data, with some transformation and theoretical interpretation.	Moderate concerns regarding the partial relevance of two studies assessing or designing interventions (Godziuk 2022; Lawford 2024). Additionally, all studies came from five high-income countries. Therefore, global relevance is limited/uncertain.	High confidence	High confidence due to the consistency across papers, with rich data to support the same. Some concerns re: methodology, but not considered impactful.	Janke 2012; Bunzil 2019; Cooper 2018; Craft 2015; Godziuk 2022; Janke 2015; O'Neill 2011; Toye 2018; Storm 2023; Lawford 2024;
3	Theme 3: The Stigmas Associated with Pain and Obesity<br><br>PwO and CP encountered various types of stigma, self-blame and altered sense of self-image resulting from living with pain and obesity. Including a sense of personal failure and frustration linked to perceived failure to lose weight, a sense of lack of control, isolation and altered self-image. HCPs exacerbating this stigmatisation - all colluding to this predominantly reductive impact psychosocially.	Moderate concerns regarding methodological limitations. Of the 10 studies that contributed - 1 had nil concerns, 7 had minor, 2 (Toye and O'Neill) had major concerns regarding methodological clarity. Most other concerns were related to relationship between researcher and participant.	Nil / very minor concerns regarding coherence.	Minor concerns regarding adequacy relating to self-image aspect, but very rich data with relation to self-blame and stigma. Some transformation and theoretical interpretation of evidence about self-efficacy, but more descriptive re: stigma.	Nil / very minor concerns regarding the partial relevance of two studies assessing or designing interventions (Godziuk 2022; Lawford 2024). Additionally, all studies came from five high-income countries. Therefore, global relevance is limited/uncertain.	High confidence	High confidence due to the consistency across all papers. Data is richer in 7 papers relating to stigma. Some concerns re: methodology, but not considered impactful.	Janke 2012; Bunzil 2019; Cooper 2018; Craft 2015; Godziuk 2022; Janke 2015; O'Neill 2011; Toye 2018; Storm 2023; Lawford 2024;

	Finding	Methodological Limitations	Coherence	Adequacy	Relevance	Overall assessment of confidence	Explanation of GRADE-CERQual assessment	References
3.1	Subtheme 3.1: Healthcare Stigma and the Moralisation of Pain and Body Weight.<br><br>Weight bias was frequently described, and commonly within the context of healthcare. Participants in seven of the studies highlighted what they felt to be a lack of understanding and holistic support from HCPs in terms of addressing their needs with respect to their pain and weight. They described a sense of blame emanating from HCPs for both weight and pain. Some participants described other heath conditions being attributed to weight. In one study, a participant recounted denial of treatment contingent on independently losing weight.	Moderate concerns regarding methodological limitations. Of the 8 studies that contributed - 6 had minor, 2 (Toye and O'Neill) had major concerns regarding methodological clarity. Most other concerns were related to relationship between researcher and participant.	Nil / very minor concerns regarding coherence.	Minor concerns regarding adequacy relating to healthcare aspect in 3 studies, but very rich data with relation to moralisation of body weight. Some transformation and theoretical interpretation of evidence about healthcare, but more descriptive re: body weight stigma.	Nil / very minor concerns regarding the partial relevance of one study assessing or designing interventions (Godziuk 2022). Additionally, all studies came from five high-income countries. Therefore, global relevance is limited/uncertain.	High confidence	High confidence due to the consistency across all papers. Data is richer relating to stigma. Some concerns re: methodology, but not considered impactful.	Janke 2012; Bunzil 2019; Cooper 2018; Craft 2015; Godziuk 2022; Janke 2015; O'Neill 2011; Toye 2018
3.2	Subtheme 3.2: Social Isolation and Progressive Withdrawal<br><br>Participants also described elements of social isolation and progressive withdrawal. They perceived a lack of understanding from family at times and explained how living with comorbid CP and obesity negatively affected engagement with many aspects of daily life which cumulatively had a progressively reductive impact psychologically and socially.	Moderate concerns regarding methodological limitations. Of the 10 studies that contributed - 1 had nil concerns, 7 had minor, 2 (Toye and O'Neill) had major concerns regarding methodological clarity. Most other concerns were related to relationship between researcher and participant.	Nil / very minor concerns regarding coherence.	Minor concerns regarding adequacy relating to self-image aspect, but very rich data with relation to self-blame and stigma. Some transformation and theoretical interpretation of evidence about self-efficacy, but more descriptive re: stigma.	Nil / very minor concerns regarding the partial relevance of two studies assessing or designing interventions (Godziuk 2022; Lawford 2024). Additionally, all studies came from five high-income countries. Therefore, global relevance is limited/uncertain.	High confidence	High confidence due to the consistency across all papers. Data is richer in some papers re: social stigma and richer in others re: isolation. Some concerns re: methodology, but not considered impactful.	Janke 2012; Bunzil 2019; Cooper 2018; Craft 2015; Godziuk 2022; Janke 2015; O'Neill 2011; Toye 2018; Storm 2023; Lawford 2024;
4	Theme 4: Food as a Complex and Frustrating Pathway to Health<br><br>Participants described altered relationships with food and perceived food as key to improving health and managing both their pain and obesity. Participants expressed a desire for changes to dietary habits, but frustration with perceived failure at sustainable change despite multiple previous attempts.	Moderate concerns regarding methodological limitations because of the 9 studies that contributed - 1 had nil concerns, 6 had minor, 2 (Toye and O'Neill) had major concerns re methodology / clarity. Most concerns were re: relationship researcher and participant.	Nil / very minor concerns regarding coherence.	Nil / minor concerns. Well-supported by more than half of the included studies and richest data came from most methodologically robust studies. Data rich with little transformation and thick contextual description.	Minor concerns regarding relevance as all studies came from 5 high-income countries. Therefore, global relevance is limited/uncertain.	High confidence	High confidence due to the consistency across all included papers. Data is richer relating to perceived frequent failure at attempted change. Some concerns re: methodology, but not considered impactful.	Janke 2012; Cooper 2018; Craft 2015; Godziuk 2022; Janke 2015; O'Neill 2011; Toye 2018; Storm 2023; Lawford 2024;

	Finding	Methodological Limitations	Coherence	Adequacy	Relevance	Overall assessment of confidence	Explanation of GRADE-CERQual assessment	References
4.1	Subtheme 4.1: Coping with Pain Through Food<br><br>Altered food habits in the context of pain, in relation to food choices and consumption (volume and quality) as a direct comfort-seeking response to pain and as an indirect consequence of pain. Pain disrupting food habits and daily routines - increased fatigue with an inverse ability to plan and prepare food, opting for foods that were faster and easier to prepare - hedonistic eating also represented.	Moderate concerns regarding methodological limitations because of the 9 studies that contributed - 1 had nil concerns, 6 had minor, 2 (Toye and O'Neill) had major concerns re methodology / clarity. Most concerns were re: relationship researcher and participant.	Nil / very minor concerns regarding coherence.	Nil / minor concerns. Well-supported by more than half of the included studies and richest data came from most methodologically robust studies. Data rich with a little transformation / interpretation of pain aspect with thick contextual description.	Minor concerns regarding relevance as all studies came from 5 high-income countries. Therefore, global relevance is limited/uncertain.	High confidence	High confidence due to the consistency across all papers. Data is richer relating to coping using food concept. Some concerns re: methodology, but not considered impactful.	Janke 2012; Cooper 2018; Craft 2015; Godziuk 2022; Janke 2015; O'Neill 2011; Toye 2018; Storm 2023; Lawford 2024;
4.2	Subtheme 4.2: Challenges Managing Nutrition and Dietary Habits<br><br>The challenge of the future, with food as a part of daily routines due to previous difficulty maintaining habits and motivation for routines perceived as healthful. Scepticism regarding practicality of altering food habits in the medium to long term and in the context of socialising and relationships - a sense of doubling the sacrifice and isolation further to those already experienced (i.e. not being able to 'indulge' in the food, but also not being able to have the same freedom of choice or experiences as others).	Moderate concerns regarding methodological limitations because of the 5 studies that contributed, all had minor concerns regarding methodology. Most other concerns related to relationship between researcher and participant.	Minor concerns regarding coherence. Some slight contradiction between studies (particularly regarding LCDs), however this is represented in the theme.	Moderate concerns regarding adequacy as only supported by half of the studies and the data was relatively thin, with some transformation and interpretation.	Minor concerns regarding relevance as all studies came from five high-income countries. Therefore, global relevance is limited/uncertain.	Moderate confidence	Moderate confidence overall due to poor data richness, absence of supportive data in other papers, and moderate concerns regarding the methodological rigour, adequacy and relevance of some of the papers that contributed to this finding.	Janke 2012; Craft 2015; Godziuk 2022; Janke 2015; Lawford 2024;

Supporting Information 5: Themes and Direct Participant Quotes
This provides direct quotes from participants, used in the synthesis of each of the themes and subthemes for this review.

**Supporting Information 5**: Themes and Direct Participant Quotes	
Theme	Quote (Source)
Theme 1: A Predominant Bio-Mechanical Understanding of Pain	"It's bone on bone, the cartilage is gone in it." (Janke, 2012)<br><br>"I keep going because I'm feeling good and…then I feel better mentally because I'm getting it done. But then I pay for it the next 5 days." (Craft, 2015)<br><br>"One day I was walking good, the next day bang…the bone was catching on bone. You can feel it actually grinding." (Janke, 2012)<br><br>"I haven't got the cartilage there, so [the physiotherapy] can't do much about that … They can't replace my cartilage: I've got to put the cushion back into my knee," (Bunzil, 2019)<br>"Well what's the point in trying to do something when something's worn out? I believe in nuts and bolts; if something's worn out, you pull it out and put a new part in." (Bunzil, 2019)<br>"One of the worst parts about it is that if I was more active, I could lose a bit of weight and take weight off [the knee]. But you're buggered because you can't do something as simple as walk down the street." (Bunzil, 2019)<br><br>"now I just feel as though it's like bone on bone and they won't do anything about it unless I lose weight (laughs) so I'm in a little.. .." (Cooper, 2018)<br><br>"If I keep going the way I am going, it's just going to get worse. It will just rub, rub away," (Bunzil, 2019)<br><br>"Look, it's wear and tear ... I expected this, I'm a hard worker," Participant 15, man, 79 years old; "I knew I was going to get it because every second person that works in the yard has got arthritis because of the way we work," (Janke, 2012)<br><br>"Wear and tear, swelling, moving it, the left one's...she, the doctor said to me "it's pretty shot" it's, it's like in bits really, but the right ones actually attached itself to the top of my eh one of my bones, she didn't say which one, but one of my leg bones" (Cooper, 2018)
	"I think it just puts all that upper body weight onto like one little joint, which on one knee is virtually non-existent and on the other one is attached to me bone, you know so....I'm sure it's making it much worse. I'm sure it's em wearing it away. I think it is, I really do" (Cooper, 2018)

	"I've worked out that I think the weight does have a lot to do with it, because em, obviously I eh I must have a nerve trapped or something but, an' then I think is it trapped because there is pressure on me hips an' everything to like move me weight around. 'Cos like I said me knee has-, burns sometimes but it has, em it doesn't burn as much since I've been losing the weight " (Cooper, 2018)
Subtheme 1.1:<br>Healthcare Professionals' Language Shapes Understanding	"They've shown me the pictures of the inside of my knees, it is literally just two round circles— balls—with nothing on them." (Janke, 2012)<br><br>"Well, I was a little disturbed with it at first …he says "that's [obesity] your problem." He said, "I can't do anything with it. All I am interested in is getting your knees fixed." He says, "you know what you are. I don't have to tell you that" (Janke, 2015)<br><br>"I've got a collapsing spine," While Emily was not surprised when she developed arthritis in her knees, as she had already been warned that this was likely to occur: "it must be about ten year ago, something like that. An' I always remember the girls sayin', the nurse sayin'- when I went for ma physio [for back pain] that the next thing to go would probably be ma knees" (Cooper, 2018)<br><br>"It's a new doctor that's em come in an' he said "oh we'll send you for an MRI to see whether or not there's any, em your discs are crumbling or it, its just a trapped nerve" (Cooper, 2018)<br><br>"You could start off with just moving enough to try and help your joints, but even the doctors I went to […], they told me unless I lose weight, they're not going to help me. This is exactly what I was told." (Godziuk, 2021)<br><br>"I've just been waiting, putting up with the pain, because all the doctors say I'm too young. But everyone in my family dies before 70. So, what, am I going to live for the rest of my life in pain? The x-rays clearly show that there's no cartilage in my knee" (Janke, 2012)<br><br>"Just the pain, the pain is like the thing that's saying you know you have to get this weight off or you're just gonna be in pain all the time and I don't go back to the doctors 'cos they'll just tell me the same thing.. ..So there's only one person who can do it and it's me.. . yeah" (Cooper et al., 2018)
Subtheme 1.2:<br>Seeking '*Safe*' Exercise:<br>Navigating Fear of Pain and Damage	"I can sw, swimming seems good because it takes the weight off it, so that's good" (Cooper, 2018)<br><br>"Learning where I need to start is most important for me. Learning how to do something and be confident is also important. […] I worry about doing exercise that a skinny little thing would put me through. This is done by someone who knows the issues posed by obesity." (Godziuk, 2021)<br><br>"You could start off with just moving enough to try and help your joints, but even the doctors I went to […], they told me unless I lose weight, they're not going to help me. This is exactly what I was told." (Godziuk, 2021)<br><br>"and when you do well, 'cos we're recording the walking so we know how long and the, the distance, it makes you think "Oh, actually I can maybe do half a mile more by the end of this week" and I think that helps, gives you something to aim for." (Cooper et al., 2018)

	"Just the pain, the pain is like the thing that's saying you know you have to get this weight off or you're just gonna be in pain all the time and I don't go back to the doctors 'cos they'll just tell me the same thing.. ..So there's only one person who can do it and it's me.. . yeah" (Cooper, 2018)<br><br>"One of the worst parts about it is that if I was more active, I could lose a bit of weight and take weight off [the knee]. But you're buggered because you can't do something as simple as walk down the street." (Bunzil, 2019)<br><br>"I keep going because I'm feeling good and...then I feel better mentally because I'm getting it done. But then I pay for it the next 5 days." (Craft, 2015)<br><br>"I kayak every week, I play golf every week…And then I was doing my exercises that the physio gave me, and I was doing them probably, on average, once a week. But my knee's been so good the last couple of months I've really stopped doing that as well, so yeah." (Lawford et al., 2023)<br><br>"After the program finished you know, it wasn't hard to maintain it, and I still do a lot of walking and you know I've been to some strength training and stuff like that and actually I'm doing running and that was my aim, to be able to run." (Lawford et al., 2023)<br><br>"I guess also because my knee's feeling really good, and I suppose I've lost a bit of the drive to lose all that did put on a couple of kilos. I'm about 84 and a half at the moment, and so I've been very happy. It's not been as difficult as I thought." (Lawford et al., 2023)<br><br>"'Exercise, nutrition' automatically that says, 'Eat less, move more'. That's what that says because fat people have heard that all their lives, right? […] I honestly think we need to get rid of that word 'exercise'. You could talk about in terms of mobility." (Godziuk, 2021)<br><br>"Ellie found some forms of exercise resulted in pain and swelling of her joints, and considered cycling as a safe form of exercise: 'I was quite up f'for gettin' a bike 'cos I thought "well that'll ease the pressure on ma knees an' ma ankles if I can cycle." However, when she went to buy a bike, concern that she was too heavy for the tyres resulted in her leaving the shop without buying or even approaching a sales assistant, who may have been able to help. Her embarrassment about her size was evident in the way she paused and laughed when she said "there wasn't a tyre that could... take ma weight ((laughing)) on any of the bikes so.. .." (Cooper, 2018)
Theme 2:<br>Catch 22:<br>Vicious Cycle of Pain and Obesity	"It is a vicious circle of.. .. . . y'know tryin' to lose weight, tryin' to.. ... y'know not be in pain, tryin' to exercise and then be in pain.... . ..y'know" (Cooper, 2018)<br><br>"It seems like you might be always chasing your tail a little bit with fibro." (Craft, 2015)<br><br>"Just dealing with the medical side of things, you add stress. Most stress affects weight. Depression affects weight. Pain affects weight. Medication affects weight." (Craft, 2015)<br><br>"It [pain] makes it [weight-loss] more difficult in the sense that it feeds right into my procrastination and I don't do walking because that's one of the exercises that I used to be very good about, was walking….And now, I think walking two blocks, it's all changed…I've gotten heavier and I've gotten lazier." (Janke, 2012)<br><br>"When you can't use your body, you get frustrated and restless [. .]. When I am in such a situation and want to break the pattern, it happens that I go out and smoke a cigarette." (Storm et al., 2023)<br><br>"You got to lose some weight.' [to have less pain] The first guy I told you about, he handed me a sheet that was 1200 calories a day. He said 'Follow this; you'll lose weight. Don't come back here if you haven't lost 20 pounds.' The first month, I lost 20 pounds, the next month I only lost 18 pounds, and he just tore me apart …" (Janke, 2015)

	"I've always attributed my problem to my weight and I figured if I lost weight, it would relieve the pressure. So I really haven't focussed on my knee, on finding a programme for arthritis. I always thought it'd be the weight that would help my walking, so I focussed on my weight more, if that makes any sense." (Godziuk, 2021)
Subtheme 2.1:<br>Pain and Obesity:<br>Both Motivators and Barriers	"It is a vicious circle of.. .. . . y'know tryin' to lose weight, tryin' to.. ... y'know not be in pain, tryin' to exercise and then be in pain.... . ..y'know" (Cooper, 2018)<br><br>"It [pain] took away the desire to lose weight…I was just like, 'Oh, forget it. I don't want to worry about that right now." (Janke, 2012)<br><br>"Just dealing with the medical side of things, you add stress. Most stress affects weight. Depression affects weight. Pain affects weight. Medication affects weight." (Craft, 2015)<br><br>"I kayak every week, I play golf every week…And then I was doing my exercises that the physio gave me, and I was doing them probably, on average, once a week. But my knee's been so good the last couple of months I've really stopped doing that as well, so yeah." (Lawford et al., 2023)<br><br>"he said "You've walked all the ways to so and so, you've been running". He said "don't you realise". I'm like "I, no" and this was like.. like em last year when I was working you see so that walking in the [workplace] had helped, right, whereas.. cos I've been out of work from November and its been cold and I've got snuggled in the house I haven't bothered you see" (Cooper, 2018)<br><br>"I don't want anything I do at this stage to make it worse which is why I do quite a bit of walking in the swimming pool because then at least the buoyancy helps you along" (Cooper, 2018)<br><br>"we're going to re-join the swimming baths I'm going to go again, where we go every morning because I quite like swimming, swimming doesn't seem to put the pressure on my joints 'an I can go at me own pace" (Cooper, 2018)<br><br>"Just the pain, the pain is like the thing that's saying you know you have to get this weight off or you're just gonna be in pain all the time and I don't go back to the doctors 'cos they'll just tell me the same thing.. ..So there's only one person who can do it and it's me.. . yeah" (Cooper, 2018)<br><br>"You got to lose some weight.' [to have less pain] The first guy I told you about, he handed me a sheet that was 1200 calories a day. He said 'Follow this; you'll lose weight. Don't come back here if you haven't lost 20 pounds.' The first month, I lost 20 pounds, the next month I only lost 18 pounds, and he just tore me apart …" (Janke, 2015)<br><br>"When you can't use your body, you get frustrated and restless [. .]. When I am in such a situation and want to break the pattern, it happens that I go out and smoke a cigarette." (Storm et al., 2023)<br><br>"“I've always attributed my problem to my weight and I figured if I lost weight, it would relieve the pressure. So I really haven't focussed on my knee, on finding a programme for arthritis. I always thought it'd be the weight that would help my walking, so I focussed on my weight more, if that makes any sense." (Godziuk, 2021)<br><br>"One of the worst parts about it is that if I was more active, I could lose a bit of weight and take weight off [the knee]. But you're buggered because you can't do something as simple as walk down the street." (Bunzil, 2019)
Subtheme 2.2:<br>The Impact of<br>Negative Emotions on Pain	"I'm taking antidepressants which add to weight gain. There was a doctor that said, 'I treat all these patients that take antidepressants and there is a way to lose weight taking them.' I tried that diet and it's a 1400 calorie diet. Well, you can't get up and function all day when you are eating a 1400 calorie diet for the next 6 months. She said, 'This is really easy. You just eat this 1400 calorie diet.' I don't know where to get the real information. I don't know where to go to get a plan that will work for me. And all of the things

	that I get exposed to, I'm sorry, I'm getting emotional, that I try, I seem to fail. I just can't seem to make it work. Either I'm getting bad information or I'm not trying hard enough.” (Craft, 2015)<br>“I believe that the depressed pain which I didn’t recognize as pain then, disappeared [after weight-loss]. But then as soon as I was off it and started gaining it back, it started all over again. And as years went by, the physical things happened, the arthritis along with the weight made it worse.” (Janke, 2012)<br><br>“The depression just makes it all that much worse. I think the treatments for pain will work better if we could get depression under control.” (Janke, 2012)<br><br>“I found I was losing weight finally and dealing with my fibromyalgia but I was in a lot of pain and so they put me on medications and now, I'm gaining weight again with the medications.” (Craft, 2015)<br><br>“My husband is like, ‘Really? You don't want to get up off the couch?’ And I say, ‘Honey, I just can't function.” (Craft, 2015)<br><br>“‘It has been a difficult transition from the very depressed, shuffling, very obese excuse of a 60-something man… Never mind the pain, depression and acute swelling, I hated it, this was even before I had taken my coat off. All of the phone calls, letters and meetings would go to waste, yet alone all of the offers put in by others behind the scenes. Within 5 minutes the nurse specialist along with the ward manager came to advise me that they had found an alternative bed, by myself, in an unoccupied ward, this to me was heaven as I did not want other people to see my legs, I had become very self-conscious..” (O’Neill, 2011)<br>“I love to pick up whatever I want to eat, fix it and eat it. But, I know now that I can't because if I do, I'm going to keep getting bigger and bigger and bigger. And, that right there, in itself, plays the emotional part. Because when I'm standing in the bathroom, getting ready to get in the shower, I look at myself and I cry. And I think, ‘How did I get here?’ So, it all plays a role but then I'm hurting, so I don't want to go downstairs and do exercises… But my emotional comes back on the pain and if I was smaller, maybe I wouldn't hurt as bad to where I could get in 30 minutes every other day of exercise.” (Craft et al., 2015)<br>“I lost all my discipline for my physical being, and I just acting like I didn’t care no more…like haywire and I would eat, where someone would eat a normal hot dog and that would be enough for them, and they would be satisfied, it took me three or four to get satisfied. I still don’t feel satisfied. I have to stop myself from continuing and eating.” (Janke, 2012)
Theme 3:<br>The Stigmas Associated with Pain and Obesity	“I think it’s just mind over matter what you want really isn’t it … I look at people and I see them the size of a bus and I just couldn’t be like that … Its laziness and their attitude to everyday life…trouble is everybody has got so lazy haven’t they … I just think people are lazy cos there are no predators around you see.” (Toye et al., 2018)<br><br>“In no way can I be hungry…I use food to soothe myself, but it really doesn’t because once I’ve overeaten I say ‘Well, there. You did it again.’ I sabotage myself.” (Janke, 2012)<br><br>“I love to pick up whatever I want to eat, fix it and eat it. But, I know now that I can't because if I do, I'm going to keep getting bigger and bigger and bigger. And, that right there, in itself, plays the emotional part. Because when I'm standing in the bathroom, getting ready to get in the shower, I look at myself and I cry. And I think, ‘How did I get here?’ So, it all plays a role but then I'm hurting, so I don't want to go downstairs and do exercises… But my emotional comes back on the pain and if I was smaller, maybe I wouldn't hurt as bad to where I could get in 30 minutes every other day of exercise.” (Craft et al., 2015)<br><br>“Ellie found some forms of exercise resulted in pain and swelling of her joints, and considered cycling as a safe form of exercise: ‘I was quite up f’for gettin’ a bike ‘cos I thought “well that’ll ease the pressure on ma knees an’ ma ankles if I can cycle.” However, when she went to buy a bike, concern that she was too heavy for the tyres resulted in her

	leaving the shop without buying or even approaching a sales assistant, who may have been able to help. Her embarrassment about her size was evident in the way she paused and laughed when she said "there wasn't a tyre that could... take ma weight ((laughing)) on any of the bikes so.. .." (Cooper, 2018)<br><br>"My husband is like, 'Really? You don't want to get up off the couch?' And I say, 'Honey, I just can't function." (Craft, 2015)<br><br>"He said "You've walked all the ways to so and so, you've been running". He said "don't you realise".I'm like "I, no" and this was like.. like em last year when I was working you see so that walking in the [workplace] had helped, right, whereas..cos I've been out of work from November and its been cold and I've got snuggled in the house I haven't bothered you see." (Cooper, 2018)<br>"I lost all my discipline for my physical being, and I just acting like I didn't care no more…like haywire and I would eat, where someone would eat a normal hot dog and that would be enough for them, and they would be satisfied, it took me three or four to get satisfied. I still don't feel satisfied. I have to stop myself from continuing and eating." (Janke, 2012)
Subtheme 3.1:<br>Healthcare Stigma and the Moralisation of Pain and Body Weight	"I'm tired of 'it's in your head, you're too lazy' and then the doctors that don't want to take you on because you are the problem patient." (Craft et al., 2015)<br><br>"I'm losing weight and gaining weight and losing... I'm staying the same weight basically... she gave me the impression it was like my fault, you know that I just wasn't trying and I'm thinking well I am really but I am a little bit hampered by how little I'm starting to be able to walk, how much pain I was in" (Cooper, 2018)<br><br>"You got to lose some weight.' [to have less pain] The first guy I told you about, he handed me a sheet that was 1200 calories a day. He said 'Follow this; you'll lose weight. Don't come back here if you haven't lost 20 pounds.' The first month, I lost 20 pounds, the next month I only lost 18 pounds, and he just tore me apart …" (Janke, 2015)<br><br>"I think it's just mind over matter what you want really isn't it … I look at people and I see them the size of a bus and I just couldn't be like that … Its laziness and their attitude to everyday life…trouble is everybody has got so lazy haven't they … I just think people are lazy cos there are no predators around you see." (Toye et al., 2018)<br><br>"It [pain] makes it [weight-loss] more difficult in the sense that it feeds right into my procrastination and I don't do walking because that's one of the exercises that I used to be very good about, was walking….And now, I think walking two blocks, it's all changed…I've gotten heavier and I've gotten lazier." (Janke, 2012)<br><br>"You could start off with just moving enough to try and help your joints, but even the doctors I went to […], they told me unless I lose weight, they're not going to help me. This is exactly what I was told." (Godziuk, 2021)<br><br>"I understood that the general opinion among the staff was that I would last 2 days, and I believe it was actually planned to pack up my bits and pieces for transportation. Depression at this time was at its height, I wanted to be in the security of my home with tea-making facilities when I wanted them, where I could smoke, play with my pets, use my computer; the list of excuses grew longer." (O'Neill, 2011)<br><br>"I knew that when I went to see the surgeon he'd probably say, 'lose a bit of weight' so I was trying to pre-empt that," Participant 23, man, 59 years old" (Bunzil, 2019)

	“Learning where I need to start is most important for me. Learning how to do something and be confident is also important. […] I worry about doing exercise that a skinny little thing would put me through. This is done by someone who knows the issues posed by obesity.” (Godziuk, 2021)<br><br>“I’m tired of ‘it’s in your head, you’re too lazy’ and then the doctors that don’t want to take you on because you are the problem patient.” (Craft, 2015)
	“Well, I was a little disturbed with it at first …he says “that’s [obesity] your problem.” He said, “I can’t do anything with it. All I am interested in is getting your knees fixed.” He says, “you know what you are. I don’t have to tell you that” (Janke, 2015)
Subtheme 3.2:<br>Social Isolation and<br>Progressive Withdrawal	“My husband is like, ‘Really? You don't want to get up off the couch?’ And I say, ‘Honey, I just can't function.” (Craft, 2015)<br><br>“I am 70 years old I have worked my guts out all my life and retired and I want to have some enjoyment. Do I really want to be going on a bloody diet? … I mean you come round here on a summer’s day and we get the chairs out and the umbrella up… And it’s so pleasant when the weather is nice … To sit out there without a glass of wine is criminal to me. I think if I can’t have a bottle of wine at my age… If I didn’t eat and I had to go and not have a drink I think I would get quite depressed.” (Toye 2018)<br><br>“He said “You’ve walked all the ways to so and so, you’ve been running”. He said “don’t you realise”.I’m like “I, no” and this was like.. like em last year when I was working you see so that walking in the [workplace] had helped, right, whereas..cos I’ve been out of work from November and its been cold and I’ve got snuggled in the house I haven’t bothered you see.” (Cooper, 2018)<br><br>“Ellie found some forms of exercise resulted in pain and swelling of her joints, and considered cycling as a safe form of exercise: ‘I was quite up f’for gettin’ a bike ‘cos I thought “well that’ll ease the pressure on ma knees an’ ma ankles if I can cycle.” However, when she went to buy a bike, concern that she was too heavy for the tyres resulted in her leaving the shop without buying or even approaching a sales assistant, who may have been able to help. Her embarrassment about her size was evident in the way she paused and laughed when she said “there wasn’t a tyre that could... take ma weight ((laughing)) on any of the bikes so.. ..” (Cooper, 2018)<br><br>“I don’t want to go anywhere…. I think the combination of the pain and my weight is like, I don’t want to get dressed up because I don’t have anything to wear. I get discouraged when I can’t do very much…. I gotta plan things that aren’t going to be real intense.” (Janke, 2012)<br><br>“Last night (my boyfriend) wants to run to McDonald's. So emotionally, I blow up. I'm like, ‘Seriously, you tell me that you're going to support me but you are going to bring McDonald's home and everybody is going to force feed their face with fast food that I can't eat.” (Craft, 2015)

	"I lost all my discipline for my physical being, and I just acting like I didn't care no more…like haywire and I would eat, where someone would eat a normal hot dog and that would be enough for them, and they would be satisfied, it took me three or four to get satisfied. I still don't feel satisfied. I have to stop myself from continuing and eating." (Janke, 2012)<br>"I love to pick up whatever I want to eat, fix it and eat it. But, I know now that I can't because if I do, I'm going to keep getting bigger and bigger and bigger. And, that right there, in itself, plays the emotional part. Because when I'm standing in the bathroom, getting ready to get in the shower, I look at myself and I cry. And I think, 'How did I get here?' So, it all plays a role but then I'm hurting, so I don't want to go downstairs and do exercises… But my emotional comes back on the pain and if I was smaller, maybe I wouldn't hurt as bad to where I could get in 30 minutes every other day of exercise." (Craft et al., 2015)
Theme 4:<br>Food as a Complex and Frustrating Pathway to Health	"I was always eating all the time. And I think that took away the pain really, just eating…I'm not thinking about it [pain], you know, I'm thinking wow this is good." (Janke, 2012)<br>"I just didn't have the energy, I found standing up at the stove the pain in my hips in particular and ma knees was really hard, making it really hard to stand and cook.. .we just ate rubbish" (Cooper 2018)<br>"I've probably learnt one thing, is not to overindulge in your meal; like the size of a meal. And I'm probably a lot more cognizant of the types of food I've got on my plate. So I try and avoid any real fatty items and I really only just have vegetables or salad with my dinner, and just whatever bit of meat or fish or something. So I am aware of not pigging out, if you like, on quantities of food and trying to keep them reasonably well." (Lawford, 2024)<br>"It's not practical to stand up and make a slow cook or a stew or something that takes longer time. Instead, I limit myself to do the things that I will be able to finish before the pain kicks in." (Storm, 2023)<br>"After I have been on foot the whole day and have been to work, I am immensely tired. Then it's like the whole body screams it's weary and wants energy. . fast, fast carbohydrates, candy, or chocolate. (Participant 14)" (Storm, 2023)<br>"It [pain] took away the desire to lose weight…I was just like, 'Oh, forget it. I don't want to worry about that right now.' You're just so worn out, the only thing you can think to do is give yourself a sugar kick and try to get something done." (Storm, 2023)<br>"…not doing anything gives me time to think about food… whereas, if I was outside, really working hard, not worrying about food, I would definitely go longer periods without eating." (Janke, 2012)
Subtheme 4.1:<br>Coping with Pain Through Food	"I lost all my discipline for my physical being, and I just acting like I didn't care no more…like haywire and I would eat, where someone would eat a normal hot dog and that would be enough for them, and they would be satisfied, it took me three or four to get satisfied. I still don't feel satisfied. I have to stop myself from continuing and eating." (Janke, 2012)<br>"In no way can I be hungry…I use food to soothe myself, but it really doesn't because once I've overeaten I say 'Well, there. You did it again.' I sabotage myself." (Janke, 2012)<br>"It used to be that I could do yogurt and I could do vegetables and that would all satisfy me and I wasn't thinking about anything else. But now the pain's there, I can't stand it. I really want my ice cream and I think I'll die if you take away my ice cream! [laughing]" (Janke, 2012)<br>"When you are in pain you can't do so much. . then I shall have something nice or cosily instead. Then it's easy to use food or something else, hmm. . candy. (Participant 2)" (Storm, 2023)<br>"I am doing things slowly due to my low energy level, and it makes me feel stressed. The stress drives me to eat unhealthy food. (Participant 5)" (Storm, 2023)

	“If I try to go for a walk in the forest or something, I must keep in mind that I have. . I usually call it ‘my pain energy’. . my energy volume is like five pieces of cake, which I can distribute every day. . ., for example, grocery shopping consumes one piece. (Participant 5)” (Storm, 2023)<br><br>“I just eat too much. You know, there’s hardly anything left other than eating.” (Janke, 2012)
	“I love to pick up whatever I want to eat, fix it and eat it. But, I know now that I can't because if I do, I'm going to keep getting bigger and bigger and bigger. And, that right there, in itself, plays the emotional part. Because when I'm standing in the bathroom, getting ready to get in the shower, I look at myself and I cry. And I think, ‘How did I get here?’ So, it all plays a role but then I'm hurting, so I don't want to go downstairs and do exercises... But my emotional comes back on the pain and if I was smaller, maybe I wouldn't hurt as bad to where I could get in 30 minutes every other day of exercise.” (Craft et al., 2015)
Subtheme 4.2:<br>Challenges Managing Nutrition and Dietary Habits	“I am 70 years old I have worked my guts out all my life and retired and I want to have some enjoyment. Do I really want to be going on a bloody diet? … I mean you come round here on a summer’s day and we get the chairs out and the umbrella up… And it’s so pleasant when the weather is nice … To sit out there without a glass of wine is criminal to me. I think if I can’t have a bottle of wine at my age… If I didn’t eat and I had to go and not have a drink I think I would get quite depressed.” (Toye 2018)<br><br>“I’m taking antidepressants which add to weight gain. There was a doctor that said, ‘I treat all these patients that take antidepressants and there is a way to lose weight taking them.’ I tried that diet and it's a 1400 calorie diet. Well, you can't get up and function all day when you are eating a 1400 calorie diet for the next 6 months. She said, ‘This is really easy. You just eat this 1400 calorie diet.’ I don't know where to get the real information. I don't know where to go to get a plan that will work for me. And all of the things that I get exposed to, I'm sorry, I'm getting emotional, that I try, I seem to fail. I just can't seem to make it work. Either I'm getting bad information or I'm not trying hard enough.” (Craft, 2015)<br><br>“Last night (my boyfriend) wants to run to McDonald's. So emotionally, I blow up. I'm like, ‘Seriously, you tell me that you're going to support me but you are going to bring McDonald's home and everybody is going to force feed their face with fast food that I can't eat.” (Craft, 2015)

Supporting Information 6: Guidance for Reporting Involvement of Patients and the Public (GRIPP2) Reporting Checklist

**Supporting Information 6**: GRIPP2 short form		
Section and Topic	Item	Reported on page no.
1: Aim	Report the aim of PPI in the study	Yes – page 4. Also outlined in protocol published May 2024 (Hinwood et al, 2024).
2: Methods	Provide a clear description of the methods used for PPI in the study	Yes – pages 4-5. Also outlined in protocol published May 2024 (Hinwood et al, 2024).
3: Study results	Outcomes—Report the results of PPI in the study, including both positive and negative outcomes	Yes – pages 3-5 and 18-19 (methodology and Reflexivity Statement and Public Patient Involvement).
4: Discussion and conclusions	Outcomes—Comment on the extent to which PPI influenced the study overall. Describe positive and negative effects	Yes – pages 3-5 and 18-9 (methodology and Reflexivity Statement and Public Patient Involvement).
5: Reflections/critical perspective	Comment critically on the study, reflecting on the things that went well and those that did not, so others can learn from this experience	Yes – page 18 (study strengths and limitations).

1. *PPI* patient and public involvement
